# Agricultural Risk Factors Influence Microbial Ecology in Honghu Lake

**DOI:** 10.1016/j.gpb.2018.04.008

**Published:** 2019-04-23

**Authors:** Maozhen Han, Melissa Dsouza, Chunyu Zhou, Hongjun Li, Junqian Zhang, Chaoyun Chen, Qi Yao, Chaofang Zhong, Hao Zhou, Jack A Gilbert, Zhi Wang, Kang Ning

**Affiliations:** 1 Key Laboratory of Molecular Biophysics of the Ministry of Education, Hubei Key Laboratory of Bioinformatics and Molecular-imaging, Department of Bioinformatics and Systems Biology, College of Life Science and Technology, Huazhong University of Science and Technology, Wuhan 430074, China; 2 Key Laboratory for Environment and Disaster Monitoring and Evaluation of Hubei, Institute of Geodesy and Geophysics, Chinese Academy of Sciences, Wuhan 430077, China; 3 The Microbiome Center, Department of Surgery, University of Chicago, Chicago, IL 60637, USA; 4 Argonne National Laboratory, Biosciences Division, Lemont, IL 60439, USA; 5 Marine Biological Laboratory, Woods Hole, MA 02543, USA; 6 State Key Laboratory of Water Ecology and Biotechnology, Institute of Hydrobiology, Chinese Academy of Sciences, Wuhan 430072, China

**Keywords:** Freshwater, Microbial communities, Agriculture activities, Antibiotics, Human impact

## Abstract

Agricultural activities, including stock-farming, planting industry, and fish aquaculture, can affect the physicochemical and biological characters of **freshwater** lakes. However, the effects of pollution producing by agricultural activities on microbial ecosystem of lakes remain unclear. Hence, in this work, we selected Honghu Lake as a typical lake that is influenced by **agriculture activities**. We collected water and sediment samples from 18 sites, which span a wide range of areas from impacted and less-impacted areas. We performed a geospatial analysis on the composition of **microbial communities** associated with physicochemical properties and antibiotic pollution of samples. The co-occurrence networks of water and sediment were also built and analyzed. Our results showed that the microbial communities of impacted and less-impacted samples of water were largely driven by the concentrations of TN, TP, NO_3_^−^-N, and NO_2_^−^-N, while those of sediment were affected by the concentrations of Sed-OM and Sed-TN. **Antibiotics** have also played important roles in shaping these microbial communities: the concentrations of oxytetracycline and tetracycline clearly reflected the variance in taxonomic diversity and predicted functional diversity between impacted and less-impacted sites in water and sediment samples, respectively. Furthermore, for samples from both water and sediment, large differences of network topology structures between impacted and less-impacted were also observed. Our results provide compelling evidence that the microbial community can be used as a sentinel of eutrophication and antibiotics pollution risk associated with agricultural activity; and that proper monitoring of this environment is vital to maintain a sustainable environment in Honghu Lake.

## Introduction

Water ecosystems, especially inland lakes, have suffered from eutrophication associated with increased agricultural activity comprising fish aquaculture as well as crop and livestock farming on surrounding lands [1], [2], [3]. Improperly managed agricultural activities, such as excessive and/or improper use of fertilizers and/or pesticides, can cause eutrophication, which can negatively impact biodiversity [4]. Previous studies have reported that the effect of this pollution on macro-organismal communities [4], [5] and in comparison, microbial ecology remains relatively understudied.

Agricultural pollution alters the physicochemical properties of water ecosystems [6] and changes the composition of microbial community. In particular, nitrogen and phosphorus content, water temperature, and pH can fundamentally influence the microbiome [7], [8], [9]. However, few studies have quantified the impact of organic pollutants such as herbicides and antibiotics. Determining the ecosystems resilience to such disturbance can aid conservation and help in the development of remediation strategies. There is an urgent need to develop sustainable approaches that establish a balanced relationship between the environment and agricultural production.

Antibiotics are widely utilized in livestock and fish aquaculture to promote animal growth and for the prophylactic or curative treatment of infectious disease [10], yet surface runoff of the introduction of treated sewage can introduce antibiotic pollution into local water bodies. Antibiotics inhibit microbial activity and can therefore influence biogeochemical processes in these ecosystems [11] and potentially select for antibiotic resistance mechanisms in environmental bacteria [12]. In addition, animal sewage can introduce animal-associated antibiotic resistant bacteria into these environments [13], and as such it is necessary to have better quantification of the fitness and recovery rates of these resistant microbes upon release into the environment [14].

**Figure 1:**
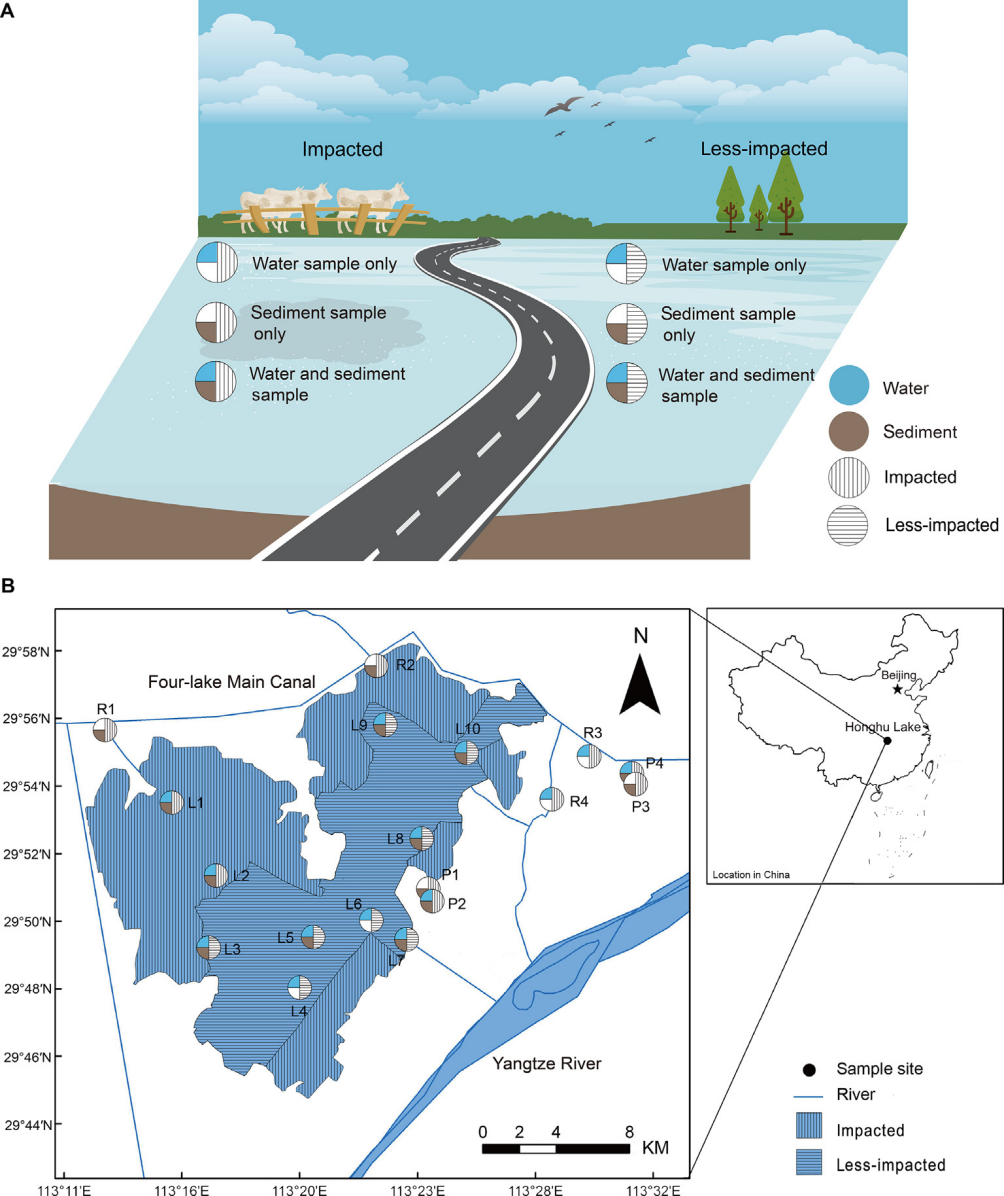
**Geographic distribution of all sampling sites in Honghu Lake** **A.** Definition of various sampling strategies. **B.** Locations of sampling sites and the distribution of the sampling medium collected at each site. The shaded segment areas with horizontal lines and vertical lines represent the less-impacted sites and impacted sites in Honghu Lake, respectively. Sites are named according to location of the sampling. L, lake; P, pond; R, river.

Honghu Lake is a large and a shallow eutrophic lake, which is located between the irrigation channel of the Four-lake main canal and the Yangzi River. Its area is about 350 km^2^ with an average depth of ∼1.5 m (Figure 1). In the last five decades, Honghu Lake has been extensively altered by flood regulation, irrigation, fish aquaculture, shipping, and water supply demands [15], [16]. Today, more than 40% of the lake area is used for large-scale aquaculture [17]. The intensive use of Honghu lake resources and the emission of sewage and other pollutants including fertilizers, pesticides, and antibiotics into the lake have led to a severe degradation of its water quality and an increase in the frequency of eutrophication events. In 2004, the Honghu Lake Wetland Protection and Restoration Demonstration Project [17] was implemented to ameliorate the negative effects of severe water pollution, and one third of the lake area has been gradually protected under this provision. Consequently, Honghu Lake represents a valuable, natural field site for investigating both the efficacy of the restoration program and the long-term effects of agricultural activities, such as the excessive application of antibiotics on water microbial communities.

This study aimed to understand the geospatial influence of pollution on the water and sediment-associated microbial communities in Honghu Lake. We performed 16S rRNA amplicon sequencing to characterize the microbial ecology, which was correlated with physicochemistry and antibiotic concentrations in these environments. This research was guided by the following scientific questions: (i) How does microbial diversity differ between water and sediment? (ii) How does microbial diversity differ between less-impacted and impacted samples in water and sediment, respectively? (iii) Which physicochemical properties and antibiotics are correlated with changes in microbial community structure? (iv) How are the co-occurrence relationships between microbiota influenced by the intensity of agricultural pollution (impacted and less-impacted)? Importantly, this baseline study aims to generate microbial biomarkers of pollution, and to identify the ecological trends that could be used to provide a sentinel of pollution events in this lake environment.

## Results

### Physicochemical and antibiotic characterization

Physicochemical characteristics and antibiotic concentrations were determined for all water and sediment samples (Figure 1, File S1, Tables S1, S2, and S3). Significant differences in pH (*t*-test, *P =* 0.0037), oxidation–reduction potential (ORP, *t*-test, *P =* 0.00068), total nitrogen (TN, *t*-test, *P =* 0.035), and ammonium nitrogen (NH_4_^+^-N, *t*-test, *P =* 0.045) were observed between water samples from impacted and less-impacted (control) sites (Table S1). Samples from impacted sites were significantly more acidic and had greater concentrations of ORP, TN, and NH_4_^+^-N when compared to less-impacted sites (Table S1). Similarly, sediment samples maintained significantly different sediment organic matter (Sed-OM, *t*-test, *P =* 0.002), sediment labile phosphorus (Sed-LP, *t*-test, *P =* 0.0335), sediment total nitrogen (Sed-TN, *t*-test, *P =* 0.0013), and sediment total phosphorus (Sed-TP, *t*-test, *P =* 0.021) levels between impacted and less-impacted sites (Table S2). Less-impacted sediment had greater concentrations of Sed-OM, Sed-LP, and Sed-TN when compared to the impacted sites (Table S2), which may largely be due to the decomposition of plant material over the preceding winter months. Between impacted and less-impacted sites, the antibiotics ofloxacin (OFL, *t*-test, *P =* 0.0079) and sulfamethoxazole (SMZ, *t*-test, *P =* 0.043) had significantly different concentrations in water samples, while sulfamerazine (SMR, *t*-test, *P =* 0.021) was significantly different in sediment samples (Table S3); in both cases concentrations were greater in impacted sites.

### Microbial diversity and community structure

A total of 28 water and sediment samples generated 4,441,405 paired-end 16S rRNA reads, which clustered into 7785 OTUs (File S1, Table S4). Microbial alpha diversity was significantly greater in sediment samples [Chao1 (*t*-test, *P =* 0.0045, Table S4) and phylogenetic diversity (PD) whole tree (*t*-test, *P* = 0.003, Table S4)]. The microbial alpha diversity in sediment samples was significantly different between impacted and less-impacted sites (*t*-test, *P =* 0.0445, Table S4). However, we observed that the alpha diversities in water samples have no significant differences.

**Figure 2:**
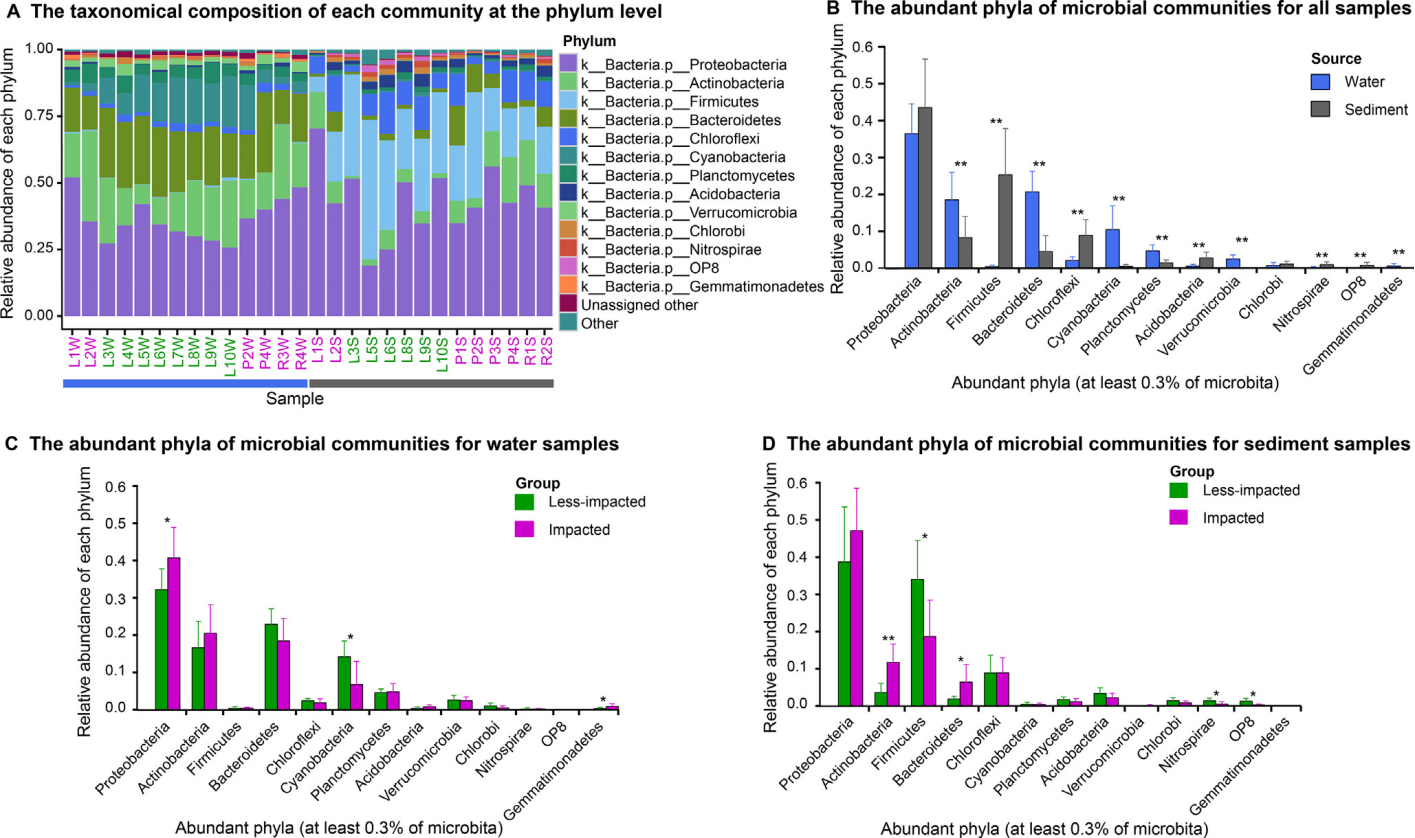
**Taxonomic composition and relative abundances of microbial taxa in water and sediment samples** **A.** Taxonomic composition of each sample at the phylum level. ‘Other’ represents all phyla not included in the top 13 phyla. Samples are named according to the sampling sites (see Figure 1B) with postfixes W and S for water and sediment, respectively. **B.** Bar plot highlighting differences between water and sediment samples at the phylum level. **C.** Bar plot highlighting differences in water samples at the phylum level between impacted and less-impacted groups. **D.** Bar plot highlighting differences in sediment samples at the phylum level between impacted and less-impacted groups. Student’s *t*-test is performed to determine significant differences between samples collected from different sampling media (water *vs*. sediment) or locations (impacted and less-impacted). ^*^P < 0.05; ^**^P < 0.01.

A total of 53 microbial phyla were identified across all samples (Figure 2A), and were differentiated between water and sediment samples (Figure 2B), and between impacted and less-impacted sites (PERMANOVA, Bray–Curtis distance, *P* < 0.01). In water samples, Proteobacteria (*t*-test, *P* < 0.05), Cyanobacteria (*t*-test, *P* < 0.05), and Gemmatimonadetes (*t*-test, *P* < 0.05) were significantly different between impacted and less-impacted sites (Figure 2C). While in sediment samples, Actinobacteria (*t*-test, *P* < 0.01), Firmicutes (*t*-test, *P* < 0.05), Bacteroidetes (*t*-test, *P* < 0.05), Nitrospirae (*t*-test, *P* < 0.05), and OP8 (*t*-test, *P* < 0.05) were significantly different between impacted and less-impacted sites (Figure 2D).

**Figure 3:**
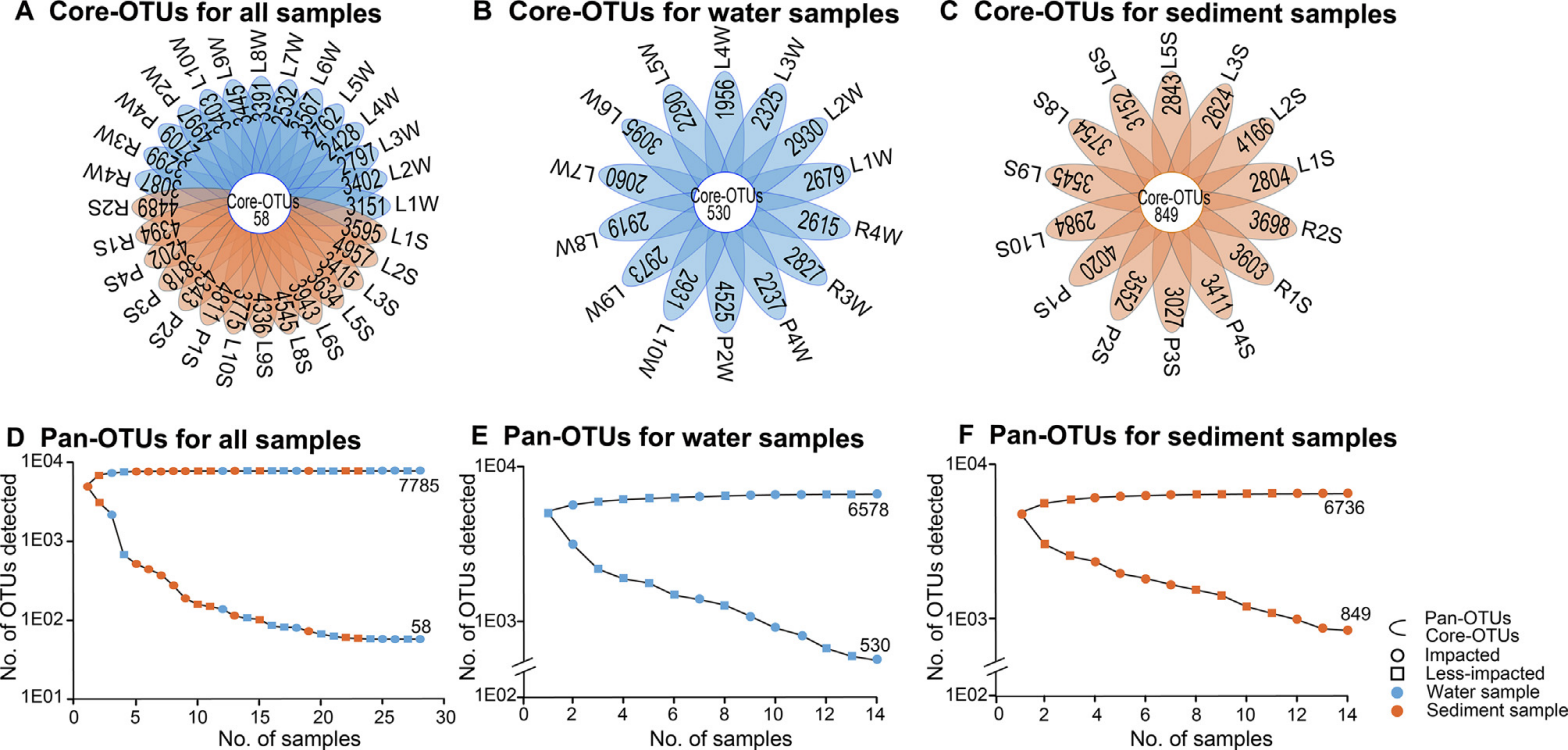
**Core-OTUs and pan-OTUs of water and sediment samples from Honghu Lake** Flower plots showing the number of sample-specific OTUs (in the petals) and core-OTUs (in the center) for all samples (**A**), all water samples (**B**), and all sediment samples (**C**). OTU accumulation curves for pan-OTUs (top) and core-OTUs (bottom) for all samples (**D**), all water samples (**E**), and all sediment samples (**F**), respectively, from Honghu Lake. Samples are named according to the sampling sites (see Figure 1B) with postfixes W and S for water and sediment, respectively.

Core-OTUs were defined as a set of OTUs that were identified in all samples analyzed, and pan-OTUs were defined as a set of OTUs that were identified in at least one sample. Core- and pan-OTUs were determined for all water and sediment samples (Table S5, Figure 3). A total of 132 core-OTUs and 7418 pan-OTUs were identified in less-impacted sites, while impacted sites maintained 201 core-OTUs and 7706 pan-OTUs (Figures S1 and S2). The core-OTUs from both the impacted and less-impacted sites were dominated by Proteobacteria, specifically *Janthinobacterium* (Tables S6 and S7), while Acidobacteria were enriched at the impacted sites (2.79% ± 1.30%, Table S6).

**Figure 4:**
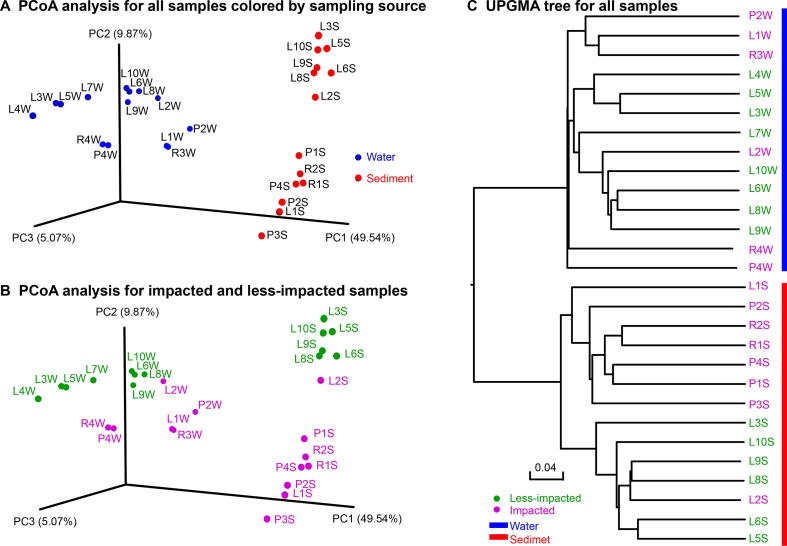
**PCoA plots and UPGMA-based clustering of water and sediment microbial communities** Unweighted UniFrac dissimilarity matrix scores for all samples were visualized in a PCoA plot to demonstrate the dissimilarity of the microbial community structure between samples by sampling medium, water *vs*. sediment (**A**) and by sampling location, impacted and less-impacted (**B**). **C.** UPGMA-based clustering tree of microbial communities using an unweighted UniFrac distance matrix. The green and pink fonts represent less-impacted and impacted groups, respectively. The blue and red bars mark water and sediment samples, respectively. Samples are named according to the sampling sites (see Figure 1B) with postfixes W and S for water and sediment, respectively.

Microbial beta diversity was further assessed by Unweighted Pair Group Method with Arithmetic Mean (UPGMA) clustering using the unweighted UniFrac distance matrix. We observed clustering by sampling medium (Figure 4A and Figure S3) and by level of agricultural activity within water and sediment samples (Figure 4B). Importantly, greater differences in beta diversity were observed between impacted and less-impacted sites in sediment samples as compared to water samples (Figure 4B and C).

### Comparison of functional properties between less-impacted and impacted groups

We observed clustering of water and sediment microbial communities based on the relative abundance of their predicted functional profiles (Figure S4) (PERMANOVA, Bray–Curtis distance, *P* < 0.0001). In water samples, functional groups including amino acid related enzymes, peptidases, oxidative phosphorylation, purine metabolism, pyrimidine metabolism, DNA repair and recombination proteins, and arginine and proline metabolism were enriched (Figure S5). Likewise, we observed an enrichment of functional groups including ribosome biogenesis, secretion system, two-component system, ABC transporters, and pyruvate metabolism in sediment samples (Figure S5). When investigating agricultural pollution risks, we observed significant differences in the relative abundances of the predicted functional profiles between impacted and less-impacted groups of water samples (PERMANOVA, Bray–Curtis distance, *P* < 0.05). For these samples, the relative abundances of DNA repair and recombination proteins (*t*-test, *P* < 0.05), purine metabolism (*t*-test, *P* < 0.05), secretion systems (*t*-test, *P* < 0.05), oxidative phosphorylation (*t*-test, *P* < 0.05), pyrimidine metabolism (*t*-test, *P* < 0.05), amino acid related enzymes (*t*-test, *P* < 0.05), and arginine and proline metabolism (*t*-test, *P* < 0.05) were significantly different between impacted and less-impacted sites (Figure S5). In contrast, we observed no significant differences in sediment functional profiles between impacted and less-impacted sites.

### Correlating physicochemical properties with microbial diversity

**Figure 5:**
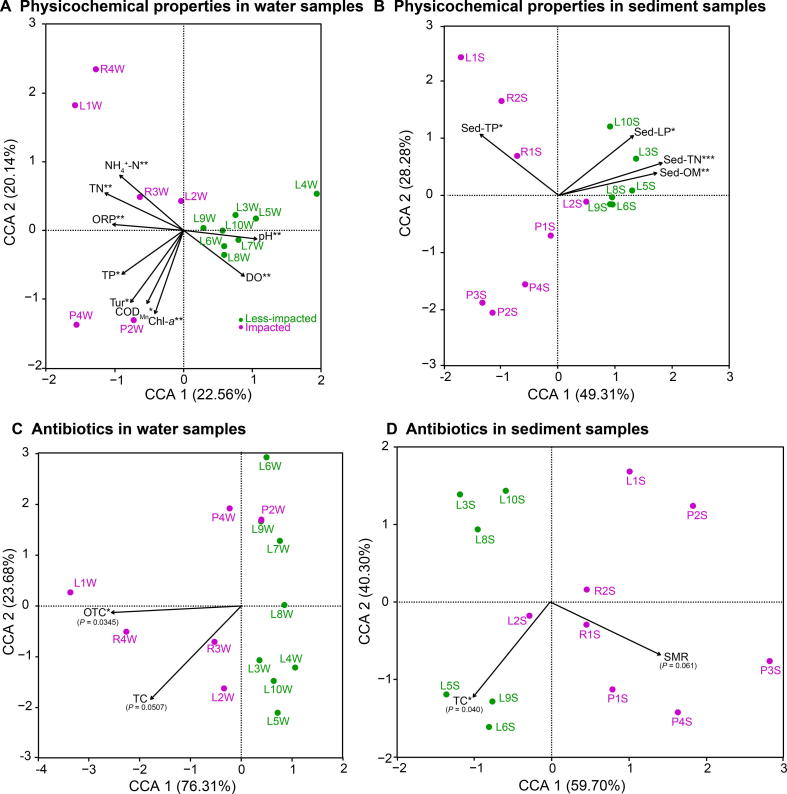
**Canonical correspondence analysis plots of physicochemical properties and antibiotic data driving water and sediment microbial community structure** Physicochemical properties of water samples (**A**) and sediment samples (**B**), as well as antibiotic data for water samples (**C**) and sediment samples (**D**) from Honghu Lake. We utilized the ‘envfit’ function with 999 permutations to reveal significant correlations between physicochemical properties, antibiotics, and microbial communities. ^*^P < 0.05; ^**^P < 0.01; ^***^P < 0.001. DO, dissolved oxygen; ORP, oxidation–reduction potential; Tur, turbidity; Chl-*a*, chlorophyll-*a*; TP, total phosphorus; TN, total nitrogen; COD_Mn_, oxygen consumption; OM, organic matter; LP, labile phosphorus; OTC, oxytetracycline; TC, tetracycline; SMR, sulfamerazine.

**Figure 6:**
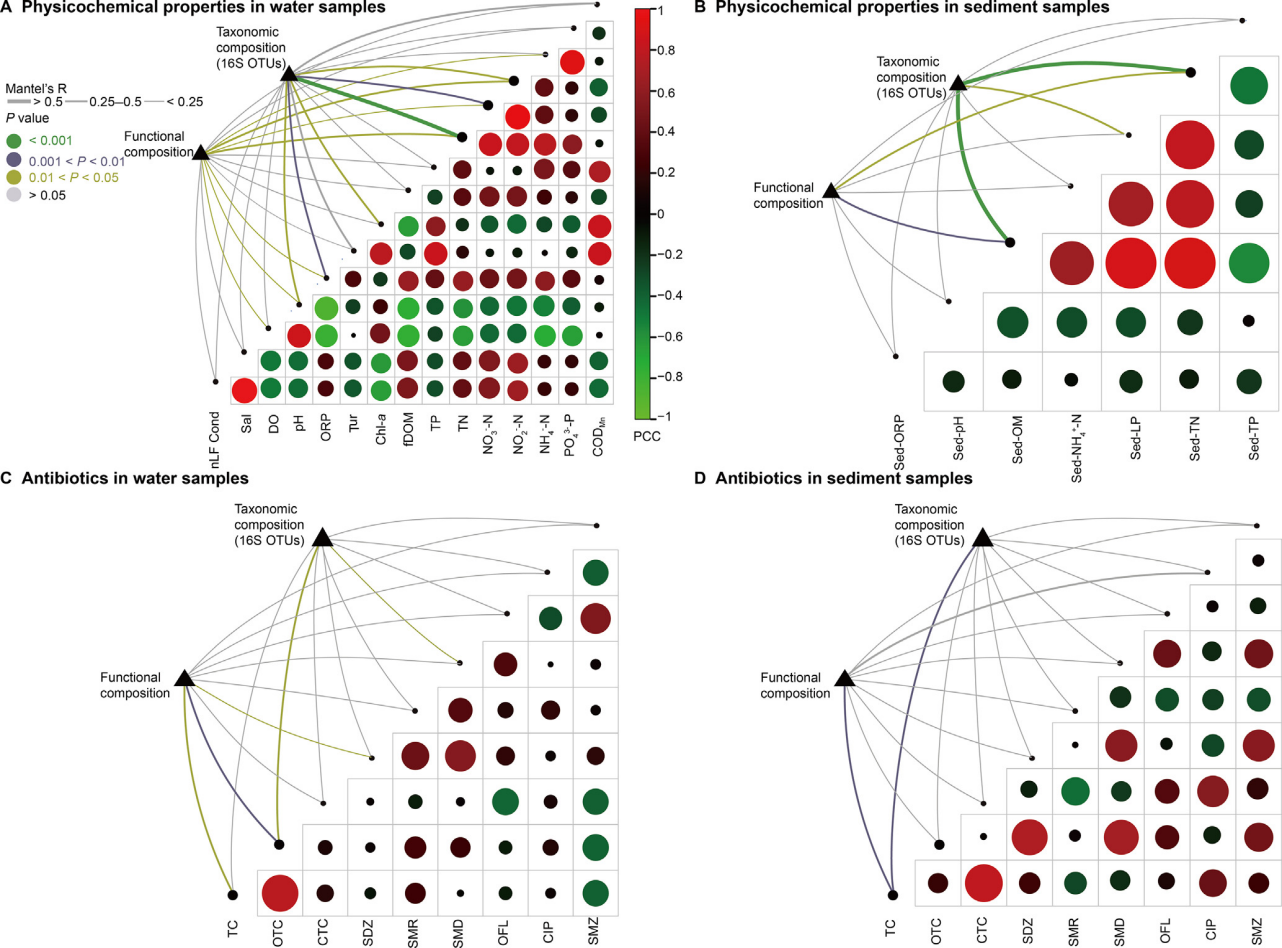
**Environmental drivers of microbial community composition in water and sediment samples** Pairwise comparison of physicochemical properties with taxonomic and functional composition data in water (**A**) and sediment (**B**) samples. Pairwise comparisons of antibiotic concentration data with taxonomic and functional composition data in water (**C**) and sediment (**D**) samples. The actual PCC values are indicated in color gradient (with green for lower PCC values and red for higher PCC values), while the absolute PCC values are indicated using circle with bigger size representing higher absolute PCC values between the two factors. The edge width represents Mantel’s *R* statistic value for distance correlation and the edge color denotes the statistical significance (*P* values) based on 9999 permutations. PCC, Pearson’s correlation coefficient; nlF Cond, temperature compensated conductivity; Sal, salinity; DO, dissolved oxygen; ORP, oxidation–reduction potential; Tur, turbidity; Chl-*a*, chlorophyll-*a*; fDOM, fluorescent dissolved organic matter; TP, total phosphorus; TN, total nitrogen; COD_Mn_, oxygen consumption; OM, organic matter; LP, labile phosphorus; TC, tetracycline; OTC, oxytetracycline; CTC, chlortetracycline; SDZ, sulfadiazine; SMR, sulfamerazine; SMD, sulfadimidine; OFL, ofloxacin; CIP, ciprofloxacin; SMZ, sulfamethoxazole.

Physicochemical properties including NH_4_^+^-N, TN, ORP, TP, turbidity (Tur), potassium permanganate index (oxygen consumption, COD_Mn_), and chlorophyll-*a* (Chl-*a*, Table S8) were significant explanatory factors that determined the observed clustering pattern of the water microbial communities at impacted sites (Figure 5A, Figure S6A), while pH and dissolved oxygen (DO) determined the water microbial community structure at less-impacted sites (Figure 5A, Figure S6A). For sediment samples, Sed-LP, Sed-TN, and Sed-OM (Table S9) were identified as significant explanatory factors shaping the observed clustering pattern at less-impacted sites and Sed-TP for impacted sites (Figure 5B, Figure S6B). Based on distance correlations and the statistical significance of Mantel’s r-statistic, water physicochemical properties including TN, ORP, nitrate nitrogen (NO_3_^−^-N), and nitrite nitrogen (NO_2_^−^-N), were strongly correlated with taxonomic and functional composition (Figure 6A). For sediment samples, Sed-OM and Sed-TN were strongly correlated with taxonomic composition (Figure 6B).

The antibiotic oxytetracycline (OTC) was the primarily explanatory factor for water microbial diversity variance at impacted sites (Figure 5C, Figure S6A). While in sediment samples, SMR was the primary factor responsible for the observed clustering of samples, including R1S, R2S, P1S, P3S, and P4S, from impacted sites (Figure 5D, Figure S6B). Mantel’s correlation assessments were also performed between antibiotic data and compositional data for water and sediment samples (Figure 6C, D, and Figure S7). The OTC antibiotic class was strongly correlated with water taxonomic and functional composition (Figure 6C), while tetracycline (TC) was strongly correlated with sediment taxonomic and functional composition (Figure 6D). The OFL antibiotic class was strongly correlated with taxonomic and functional composition in sediment samples collected from less-impacted (control) sites (Figure S8B). Additionally, COD_Mn_ and ciprofloxacin (CIP) were strongly correlated with taxonomic and functional composition in water samples collected from impacted sites (Figure S8C).

Moreover, we observed strong correlations between several OTUs, physicochemical properties, and antibiotic concentrations (Table S10, Figures S9–12). In water samples, *Bacillus flexus* (denovo 71031, Table S10) was strongly correlated with TN (*r* = 0.8675, fdr-*P* = 7.89E−5, Figure S9C), NH_4_^+^-N (*r* = 0.8958, fdr-*P* = 7.89E−5, Figure S9D), orthophosphate (PO_4_^3-^-P, *r* = 0.832, fdr-*P* = 2.58E−4, Figure S9E), and OTC (*r* = 0.8381, fdr-*P* = 3.62E−4, Figure S10B).

### Biomarker discovery

**Figure 7:**
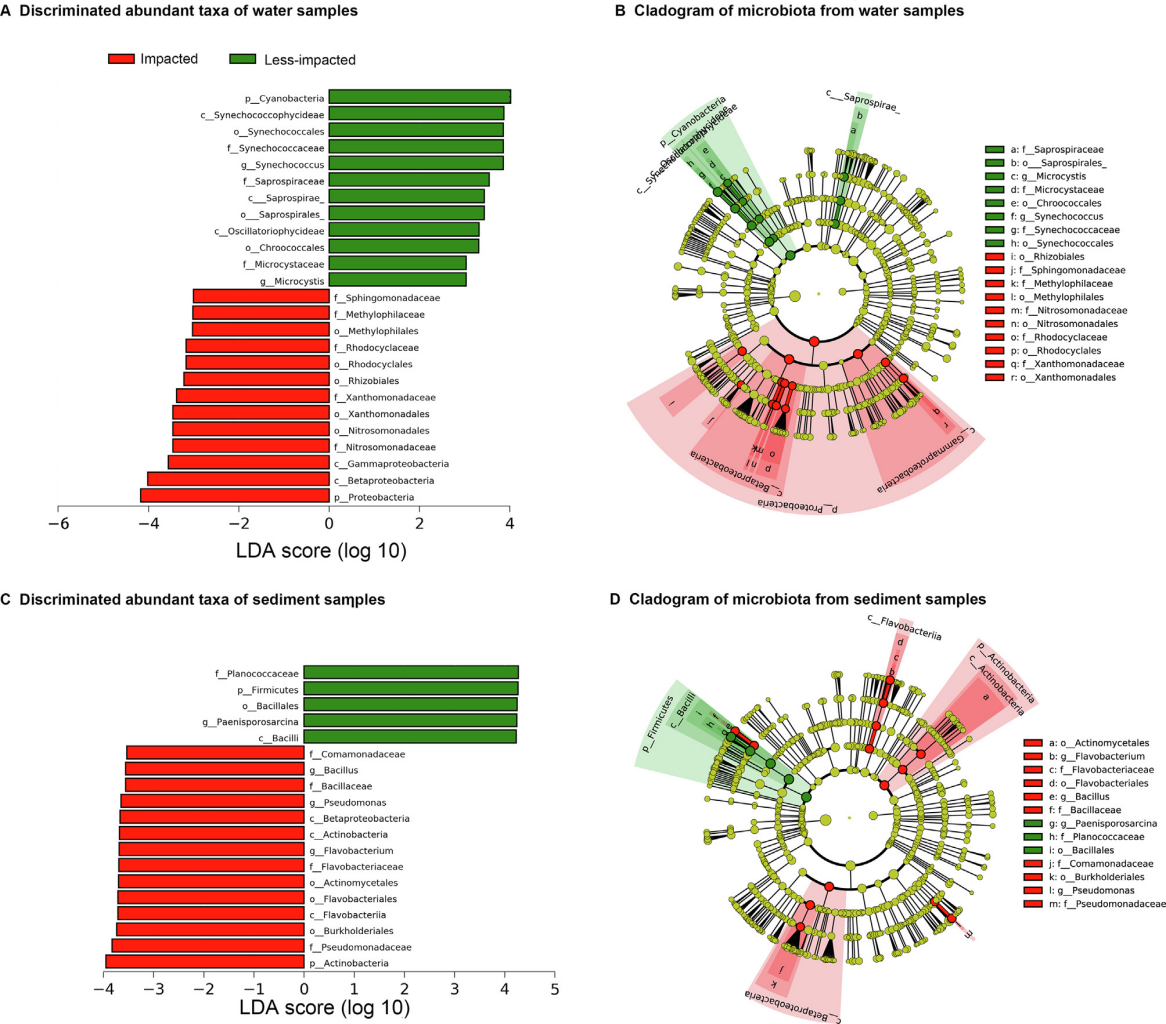
**Biomarker analysis of water and sediment microbial communities from impacted and less-impacted sites** **A.** Differentially abundant taxa of water samples. **B.** Cladogram showing the phylogenetic structure of the microbiota from water samples. **C.** Differentially abundant taxa of sediment samples. **D.** Cladogram showing the phylogenetic structure of the microbiota from sediment samples.

In water samples, the LEfSe analysis identified 13 biomarkers for impacted sites and 12 for less-impacted sites. The most differentially abundant bacteria from impacted sites belonged to the phylum Proteobacteria, class Betaproteobacteria and class Gammaproteobacteria (Figure 7A and B). These included members of the orders *Methylophilales*, *Nitrosomonadales*, and *Rhodocyclales* (Figure 7A and B). *Methylophilales* are known for their ability to metabolize methane under aerobic and microaerobic conditions [18] and *Nitrosomonadales* are significantly enriched in soils containing high concentrations of N fertilizer [19]. Water samples from less-impacted sites were overrepresented by *Oscillatoriophycideae* and *Synechococcophycideae* in *Cyanobacteria*; and *Saprospiraceae* in *Bacteroidetes* (Figure 7A and B).

In sediment samples, the LEfSe analysis reported 14 biomarkers enriched in impacted sites and 5 enriched in less-impacted sites (Figure 7C and D). Biomarkers in samples from impacted sites mainly comprised members of the phylum Actinobacteria, family Pseudomonadaceae, order Burkholderiales, and class Flavobacteriia. For sediment samples from less-impacted sites, bacteria that were differentially abundant include members of *Paenisporosarcina* genus and candidate family planococcaceae, phylum Firmicutes, order Bacillales, and class Bacilli (Figure 7C and D).

### Co-occurrence network analysis

**Figure 8:**
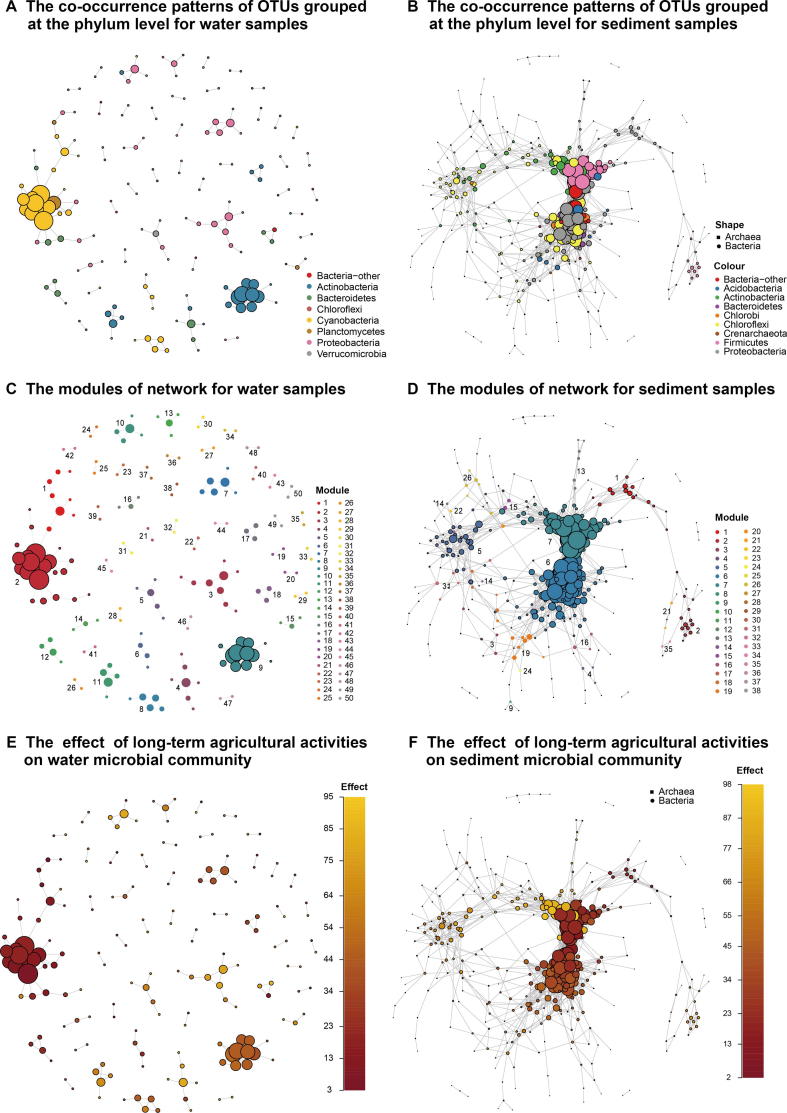
**Co-occurrence network interactions of Honghu Lake microbes in water and sediment samples** Network nodes represent OTUs with the size of each node proportional to the node degree. Edges represent positive, strong (Spearman’s *ρ* > 0.8), and significant (*P* < 0.001) interactions between OTUs. Networks of water (**A**) and sediment (**B**) samples displaying co-occurrence patterns of OTUs grouped at the phylum level. Modules were identified using the WalkTrap community detection algorithm in water (**C**) and sediment (**D**) samples. Networks of water (**E**) and sediment (**F**) samples investigating the effect of long-term agricultural activities on microbial community wherein each node is colored as a function of its relative abundance at impacted and less-impacted sites.

Co-occurrence network analysis was performed to visualize and characterize co-occurrence patterns among members of water and sediment microbial communities. The water and sediment network comprised 427 nodes and 189 edges (Figure 8A) and 443 nodes and 2877 edges (Figure 8B), respectively. The density of the water and sediment network was 0.002 and 0.023, respectively. These results suggest that the sediment microbial network was more connected than the water network. Both networks exhibited a scale-free degree distribution pattern, whereby most OTUs had low degree values and fewer hub nodes had high degree values (Figure S13).

We detected modules in water and sediment networks using the WalkTrap community detection algorithm. The modularity of the water and sediment network was 0.878 and 0.559, respectively. A total of 50 clusters with the largest membership of 22 was observed for the water network (Figure 8C). Likewise, for the sediment network we observed a total of 38 clusters with the largest membership of 111 (Figure 8D). In the sediment network, most OTUs in module 6 (111 nodes) were members of Anaerolineae of the phylum Chloroflexi and Beta-, Delta-, and Gammaproteobacteria. Additionally, most OTUs in module 7 (81 nodes) of the sediment network were members of the Planococcaceae, a family within the order Bacillales. When compared to the sediment network, we observed fewer and smaller hubs in the water network. In this network, most OTUs in module 2 (22 nodes) and module 9 (12 nodes) were members of the genus *Synechococcus* within the order Synechococcales and ACK-M1 within the order Actinomycetales, respectively.

We also examined the effect of prolonged agricultural activities on the patterns of co-occurrence network from water and sediment microbial communities, respectively. For this, each node in the water and sediment network was colored as a function of its relative abundance across samples from impacted and less-impacted (control) sites (Figure 8E and F). In both networks, we observed higher connectedness among OTUs associated with less-impacted samples as compared to those associated with samples from impacted sites. We confirmed this observation in impacted and less-impacted sediment samples by selecting OTUs that related to these sediment samples and all its edges from the overall sediment co-occurrence network to generate the sub-networks (Figure S14). We observed higher connectedness in microbes associated with less-impacted samples (measured as node degree, 3.746) as compared to those associated with samples from impacted sites (1.397).

## Discussions

The extensive application of chemical compounds such as fertilizers, herbicides, and antibiotics, can profoundly influence the cycling and accumulation of nutrients in the sediment and water column of Honghu Lake [20]. These agricultural practices can negatively impact not only the physicochemical properties, but also the biodiversity of microbial communities associated with the lake ecosystem [6]. These changes in microbial community composition can in turn affect nutrient cycling and organic matter decomposition, thus impacting overall agricultural productivity.

In our study, we analyzed water and sediment samples from Honghu Lake, assessing its microbiome, physicochemical properties, and antibiotic concentrations. We found that despite low human activity, high concentrations of Sed-LP, Sed-TN, and Sed-OM were observed at less-impacted (control) sites, probably due to the abundance of submerged plants. We speculate that the decay of these plants during winter substantially increases organic matter, total nitrogen [21], and total phosphorus [22] in sediment samples. Hence, as expected from previous research, we found that both water and sediment microbial community structure was correlated with TP and TN concentration [23], [24]. Moreover, in water samples, we observed that *Bacillus flexus* was strongly correlated with TN, NH_4_^+^-N, PO_4_^3-^-P, and oxytetracycline. More important, previous work on *Bacillus flexus* have shown that members of this species can degrade organic [25] and inorganic [26] nitrogen, thus making it a possible candidate for bioremediation in alkaline wastewater [27]. Some strains of *B. flexus* also demonstrate strong phosphorus solubilization activity [28], and others demonstrated resistance to OTC [29].

As to biomarkers in sediment samples from impacted sites, these included members of the *Hydrogenophaga* genus, belonging to Burkholderiales (Class Betaproteobacteria), which have been previously associated with agricultural activities [30]*.* Moreover, members of the genus *Pseudomonas*, belonging to family Pseudomonadaceae, can play an important role in agricultural ecosystems, particularly those associated with plant growth-promotion and disease suppression were mentioned [31].

Co-occurrence network analysis showed that Anaerolineae forms a large component of microbial communities associated with sludge wastewater treatment plants wherein they may play important roles in organic degradation [32]. The phylum Proteobacteria are known to easily metabolize soluble organic substrates [33]. Among these classes, Deltaproteobacteria, a dominant group often observed in various sediment samples, play an important role in degrading organic compounds to carbon dioxide [34]. Members of *Synechococcus* are a cosmopolitan cyanobacterium often associated with toxic algal blooms and microcystin production [35], [36]. Likewise, members of ACK-M1, in a recent study, exhibited chemotaxis toward ammonium in a water ecosystem, thus influencing nutrient cycling processes and microbial competitive interactions within this ecosystem [37]. The presence of these microbial taxa is indicative of the long-term effect of eutrophication in water environments.

## Conclusion

We analyzed the impacted sites and less-impacted sites of water and sediment samples from Honghu Lake and surrounding river and pond sites. The microbiome was analyzed in the context of variable physicochemical properties and antibiotic concentrations. There were significant differences between impacted and less-impacted (control) groups in both water and sediment samples. These differences were observed in physicochemical properties, antibiotic concentration levels, and taxonomical structure. Physicochemical properties including TN, TP, NO_3_^−^-N, and NO_2_^−^-N were the main factors driving compositional differences in water samples. Likewise, in sediment samples, Sed-OM and Sed-TN were the main factors driving differences in taxonomical composition. The antibiotics, oxytetracycline and tetracycline were identified as the main drivers of taxonomical and functional structure in water and sediment samples, respectively. As for differences between impacted and less-impacted samples, we identified 25 biomarkers within water microbial communities and 19 within sediment microbial communities. Finally, the co-occurrence network analysis revealed differences in co-occurrence patterns by sampling medium (water *vs*. sediment microbial communities) and by level of agricultural activity (impacted *vs*. less-impacted microbial communities). These results suggest that continued analyses of the composition and structure of water and sediment microbial communities in such anthropologicallyimpacted lake environments may provide valuable biomarker data to track pollution. The Honghu Lake Wetland Protection and Restoration Demonstration Project provided preliminary data that highlights the importance of monitoring biodiversity in water micro-ecosystems. Our present work allows further investigation into the impact of agricultural practices on water ecosystems and more importantly, into our ability to remediate these important ecosystems.

## Materials and methods

### Sample sites and sampling processes

To investigate the differences in microbial community structure resulting from a wide range of anthropogenic activities, a total of 14 water samples and 14 sediment samples were collected from Honghu Lake and surrounding rivers and ponds during 10–11 November 2015. Among these sites, site L1 is the entrance of inflowing river, and sites L3, L8, L9, and L10 are relatively adjacent to aquaculture district. Meanwhile, to evaluate the main source of the antibiotics of Honghu Lake, sites R1, R2, R3, and R4, which are located in four major connecting rivers of Honghu Lake and sites P1 to P4, four typical aquaculture ponds, which can swap water with Honghu Lake, were collected [38]. In keeping with the Government Protection Zone definition [17] and in taking into account the different sources of pollution at each site [38] (treated sewage, crop, livestock, and fish aquaculture), all sampling sites were categorized into two groups—namely, the impacted and the less-impacted (control) groups [17]. Sampling sites labeled L1, L2, P1, P2, P3, P4, R1, R2, R3, and R4 were classified as impacted, while sites labeled L3, L4, L5, L6, L7, L8, L9, and L10 were classified as less-impacted (Figure 1).

For water sampling, 2 L of water with a depth of 0.3–0.5 m were collected at each sampling site using a cylinder sampler. Approximately 1.5 L of sample was used for physicochemical characterization and antibiotic analysis. The remaining 500 mL of sample was size-fractionated using a 20 μm tulle and a 0.22 μm diameter pore size filter membrane (Tianjin Jinteng Experiment Equipment Co., Ltd). Microbial biomass was collected on 0.22 μm diameter pore size filter membranes. These membrane samples were stored onsite in a portable cooler with ice bags, then transported to the laboratory and stored at −80 °C until DNA extraction. For sediment sampling, ∼200 g of sediment (0–10 cm) was collected at each site and stored in a portable cooler with ice bags until its transportation to the laboratory for subsequent downstream analyses. Approximately 50 g of sediment was used for physicochemical characterization and antibiotic analysis, while the remainder was dried in an Ultra-low Freeze Dryer (Christ, German) until no further weight changes were observed. The dried sediment (0.5 g) was used for DNA extraction.

### Physicochemical characterization and antibiotic analysis

#### Physicochemical characterization

Physicochemical data were measured for all water and sediment samples (Tables S1 and S2). Physicochemical properties including water temperature (T), pH, temperature compensated conductivity (nlF Cond), DO, salinity (Sal), ORP, Tur, Chl-*a*, and fluorescent dissolved organic matter (fDOM) were measured for all water samples *in situ* by EXO2 (YSI). Additional physicochemical properties including TP, TN, NH_4_^+^-N, COD_Mn_, PO_4_^3-^-P, NO_2_^−^-N, and NO_3_^−^-N were assayed as described in previous work [39]. For sediment samples, ORP (Sed-ORP) and pH (Sed-pH) were determined using a pH/ORP portable meter (YSI). Sed-OM was determined in a muffle furnace at 550 °C [39]. Sed-LP, Sed-TP, NH_4_^+^-N (Sed-NH_4_^+^-N), and Sed-TN were measured by the NH_4_Cl extraction method, the KCl extraction method, the perchloric acid and sulfuric acid digestion method, and the Kjeldahl method, respectively [40].

#### Antibiotic analysis

Based on a report of antibiotic usage in China [41], a total of 13 antibiotics were selected for detection in water and sediment samples (Table S3). These antibiotics were classified into three groups namely: (i) sulfonamides (SAs), including sulfadiazine (SDZ), SMR, sulfamate (SFM), sulfadimidine (SMD), sulfamonomethoxine (SMM), and SMZ; (ii) fluoroquinolones (FQs), including fleroxacin (FLE), OFL, CIP, and difloxacin (DIF); and (iii) the tetracycline group (TCs), including TC, OTC, and chlortetracycline (CTC). We determined the concentration of these antibiotics in water and sediment samples using a 2695 Waters Alliance system (Milford, MA). A detailed protocol of the antibiotic extraction process was described in File S1. Of the 13 antibiotics that were quantified, nine antibiotics including TC, OTC, CTC, SDZ, SMR, SMD, OFL, CIP, and SMZ were selected for further analysis in this study.

### DNA extraction and 16S rRNA gene sequencing

DNA was extracted from water filter membranes and dried sediment using a modified hexadecyltrimethylammonium bromide (CTAB) method [42], [43], [44]. All extracted metagenomic DNA was dissolved in TE buffer and stored at −20 °C until further use.

Metagenomic DNA were quantified by using a Qubit® 2.0 Fluorometer (Invitrogen, Carlsbad, CA) and the quality of DNA was assessed on 0.8% agarose gels. Approximately 5–50 ng of DNA was used as template for amplifying the V4–V5 hypervariable region of the 16S rRNA gene of microbiota for each sample. Sequences for the paired primers are “GTGYCAGCMGCCGCGGTAA” and “CTTGTGCGGKCCCCCGYCAATTC”, respectively [24]. The sequencing library was constructed using a MetaVx™ Library Preparation kit (GENEWIZ, Inc., South Plainfield, and NJ). The ends of the 16S rDNA amplicons were added with indexed adapters by limited cycle PCR. Sequencing libraries were verified using the Agilent 2100 Bioanalyzer (Agilent Technologies, Palo Alto, CA) and quantified by Qubit® 2.0 and quantitative PCR (Applied Biosystems, Carlsbad, CA). All amplicons were sequenced on the Illumina MiSeq platform (paired-end, 2 * 300 bp). All sequencing data for the 14 water samples and the 14 sediment samples were deposited into NCBI’s Sequence Read Archive (SRA) database under the Bioproject number PRJNA352457.

### Quality control, OTU clustering, and taxonomy assignment

All 16S rRNA gene amplicons were processed according to the ensuing criteria and sequences below the set quality threshold were excluded from subsequent analyses. Firstly, paired-end reads were spliced using the ‘make.contigs’ command in mothur [45] (version 1.25.0) with default settings. We conducted the quality control to remove the low-quality reads, which contained ambiguous base calls (N), or longer than 500 bp, and those shorter than 300 bp. Putative chimeras were identified using the SILVA database [46] (Release 123) and removed using the ‘chimera.uchime’ and ‘remove.seqs’ commands in mothur. All high-quality sequences were aligned using PyNAST and dereplicated with UCLUST [47] in QIIME (Quantitative Insights Into Microbial Ecology, v1.9.1) [48]. Finally, the Greengenes database (version 13_8) [49] was used as the reference database for classifying *de novo* operational taxonomic units (OTUs) that were clustered with the 97% nucleotide identity. We set 0.001% as the threshold to filter the low-abundance OTUs and keep abundant OTUs for analysis [50].

### Microbial diversity assessment

Microbial alpha- and beta-diversity values were determined using the QIIME [48] pipeline. For alpha-diversity, rarefaction curves were drawn based on the following metrics: Observed OTUs, Chao1, PD whole tree metric, and the Shannon evenness metric [51]. For beta-diversity analysis, the final OTU table was rarefied to contain 61,088 reads per sample. Bray–Curtis, weighted and unweighted UniFrac distance metrics [52] were used to measure community similarity among samples. Microbial community clustering was arrayed by Principle Coordinates Analysis (PCoA) and visualized using Emperor [53] in QIIME. The hierarchical clustering method, UPGMA, was applied to cluster all water and sediment samples, and the clustering tree was visualized in FigTree (version 1.4.2, http://tree.bio.ed.ac.uk/software/figtree/). Permutational multivariate analysis of variance (PERMANOVA) [54] was performed on the Bray–Curtis distance matrix to compare differences in community structure.

### Functional profiling

PICRUSt (version 1.0.0-dev) [55] was used to make functional predictions based on the 16S rDNA dataset from each sample. For this, OTU-picking was performed on all quality-filtered sequence data using the ‘pick_closed_reference_otus.py’ command in QIIME. OTUs were clustered at the 97% nucleotide identity threshold using the Greengenes database. The OTU table was normalized using the ‘normalize_by_copy_number.py’ command. The normalized OTU table was used for functional prediction with the ‘predict_metagenomes.py’ script, and functional trait abundances were determined for each sample using the KEGG database (version 66.1, May 1, 2013) [56]. Finally, the predicted functional content was collapsed to level three of the KEGG hierarchy using the ‘categorize_by_function.py’ script.

### Analysis of the relationships between physicochemical properties, antibiotics, and microbial communities

Canonical correspondence analysis (CCA) was chosen and used to identify an environmental basis for community ordination, revealing relationships between microbial communities and environmental factors [57]. For this, the CCA function in R package, vegan was utilized. We utilized the ‘envfit’ function [58], [59] with 999 permutations to reveal significant correlations between physicochemical properties, antibiotics, and microbial communities. To further investigate correlations between environmental factors (including physicochemical properties and antibiotics) and OTUs, we applied a low-abundance filter to remove OTUs whose relative abundance did not exceed 0.01% in any sample (as previously reported by [60]). Similarly, for physicochemical data and antibiotics data, the values of each variable were transformed to *z*-scores [61], based on which the Pearson Correlation Coefficient between each environmental factor and each OTU was calculated. To select for significant interactions between an environmental factor and an OTU, the threshold of the *r*-value and the False Discovery Rate (FDR)-corrected *P* value of the Pearson Correlation Coefficient was set at 0.8 and 0.05, respectively.

### Analysis of environmental drivers of microbial community composition

We noted environmental drivers of microbial community composition on the basis of (i) compositional data, which include taxonomical composition (relative taxonomic abundances) and functional composition at KEGG module level three; (ii) physicochemical data; and (iii) antibiotics data. To pre-process compositional data, we applied a low-abundance filter to remove OTUs whose relative abundance did not exceed 0.01% in any sample and then log transformed the relative abundances. Likewise, for physicochemical and antibiotics data, the values of each variable were transformed to *z*-scores. Based on the Euclidean distances, we computed Mantel’s correlations between the physicochemical data and compositional data and then the antibiotics data and compositional data (9999 permutations). We obtained the results in R (version 3.3.1) and visualized it in the Adobe Illustrator (version 16.0.0). Taxonomical composition and functional composition data were correlated to each antibiotic and physicochemical property by Mantel’s tests. The distance correlations and the statistical significance of Mantel’s r statistic corresponded to edge width and edge color, respectively [60].

### Biomarker analysis

Based on their location, all water and sediment samples can be divided into two groups—impacted and less-impacted (control) groups. It is well known that the taxonomical composition of a microbial community can be impacted by local environmental variables. As a result, some bacteria might be enriched by distinctive environmental states. Linear discriminate analysis (LDA) effect size (LEfSe) [62] was used to select biomarkers in impacted and less-impacted (control) groups in water and sediment samples. Briefly, the taxa abundance table was imported into the LEfSe pipeline, and the parameters were set as follows: the alpha value for the factorial Kruskal–Wallis test [63] among classes and the *P* value for the pairwise Wilcoxon test between subclasses were both chosen to be 0.05. As to water and sediment samples, we set 3.0 and 3.5 as the threshold for the logarithmic LDA score for discriminative features, respectively.

### Co-occurrence network analysis

To reduce sparsity, we selected water and sediment OTUs that were present in at least 50% of all water and sediment samples, respectively. We then generated separate networks for water and sediment microbial communities. The co-occurrence network was constructed using the CAVNet package (https://bitbucket.org/JackGilbertLab/cavnet) in R (as previously described by [64]). Briefly, water and sediment networks were inferred using the Spearman correlation matrix with the WGCNA package [65]. In this network, co-occurring OTUs are represented by nodes and connected by edges. The network deconvolution method was utilized to distinguish direct correlation dependencies [66]. All *P* values were corrected for multiple testing using the Benjamini–Hochberg FDR-controlling procedure [67]. The cutoff of the FDR-corrected *P* value was set at 0.01. Random matrix theory-based methods were utilized to determine the cutoff of Spearman’s correlation coefficients for water (0.84) and sediment (0.81) networks. All network properties were calculated using the igraph package in R [68]. We also utilized igraph to visualize and generate water and sediment networks. The WalkTrap community detection algorithm was used to identify modules in water and sediment networks [69]. To study the effect of prolonged agricultural practices, we colored each node within the water and sediment network as function of its relative abundance at impacted and less-impacted (control) sites using the ‘plot_network_by_continuous_variable’ function in CAVNet.

## Authors’ contributions

The whole study was designed by ZW and KN. MZH, JQZ, and ZW collected samples. MZH, CYC, QY, and HZ conducted DNA extraction and sequencing. MZH, CYZ, MD, and HJL analyzed the data. MZH, CYZ, MD, HJL, JG, ZW, and KN wrote and modified the initial draft of the manuscript. All revised the manuscript.

## Competing interests

The authors declare no competing financial interests.

## Supplementary data

Supplementary File S1 **Supplementary information for ‘Agricultural Pollution Risks Influence Microbial Ecology in Honghu Lake’**

Supplementary Figure S1 **The core-OTUs and pan-OTUs of water samples and sediment samples from less-impacted group in Honghu lake** The flower plots illustrate the number of shared and specific OTUs. **A.** Flower plot showing numbers of specific OTUs (in the petals) of each water sample and sediment sample and core-OTUs (in the center) of less-impacted group samples. **B.** Flower plot showing numbers of specific OTUs (in the petal) and core-OTUs (in the center) of each water sample in less-impacted group. **C.** Flower plot showing numbers of specific OTUs (in the petal) and core-OTUs (in the center) of each sediment less-impacted group sample. **D.** OTU accumulation curves for pan-OTUs (upper) and core-OTUs (lower) for less-impacted group samples from Honghu lake. **E.** OTU accumulation curves for pan-OTUs (upper) and core-OTUs (lower) for all water samples from less-impacted group in Honghu lake. **F.** OTU accumulation curves for pan-OTUs (upper) and core-OTUs (lower) for all sediment samples from less-impacted group in Honghu lake.

Supplementary Figure S2 **The core-OTUs and pan-OTUs of water samples and sediment samples from impacted group in Honghu lake** The flower plots illustrate the number of shared and specific OTUs. **A.** Flower plot showing numbers of specific OTUs (in the petals) of each water sample and sediment sample and core-OTUs (in the center) of impacted group samples. **B.** Flower plot showing numbers of specific OTUs (in the petal) and core-OTUs (in the center) of each water sample in impacted group. **C.** Flower plot showing numbers of specific OTUs (in the petal) and core-OTUs (in the center) of each sediment impacted group sample. **D.** OTU accumulation curves for pan-OTUs (upper) and core-OTUs (lower) for impacted group samples from Honghu lake. **E.** OTU accumulation curves for pan-OTUs (upper) and core-OTUs (lower) for all water samples from impacted group in Honghu lake. **F.** OTU accumulation curves for pan-OTUs (upper) and core-OTUs (lower) for all sediment samples from impacted group in Honghu lake.

Supplementary Figure S3 **PCoA and UPGMA among microbial community taxonomical structure A.** PCoA result based on OTU relative abundances of samples using weighted Unifrac metric. The red color and blue color represented the water samples and the sediment samples, respectively. **B.** PCoA result based on OTU relative abundances of samples using weighted Unifrac metric. The different colors represented different types of samples. **C.** UPGMA result based on the weighted Unfirac metric used in PCoA. The green and pink circles represent less-impacted group and impacted group, respectively, as well as the blue and red boxes represent water and sediment samples, respectively. The clustering result helps to determine the similarity of the microbial communities between different samples. Consequently, the results showed the microbial communities was correlated with agricultural activities.

Supplementary Figure S4 **Heatmap plot showing the Kendall correlations between water and sediment samples clustered by UPGMA linkage hierarchical clustering**

Supplementary Figure S5 **Heatmap plot showing the main functional properties between water and sediment samples clustered by Spearman’s correlation coefficient and UPGMA linkage hierarchical clustering** The functional categories with relative abundances more than 0.01 in each sample were selected as main functional properties and shown in this figure.

Supplementary Figure S6 **Canonical correspondence analysis plots of environmental parameters driving water and sediment microbial community structure** Environmental parameters of water samples (**A**) and sediment samples (**B**) from Honghu lake.

Supplementary Figure S7 **Environmental drivers of microbial community composition of water samples** Pairwise comparisons of physicochemical properties and antibiotics of water samples were shown, with a color gradient denoting Pearson’s Correlations Coefficients (PCC). Taxonomic composition and functional composition were related to each physicochemical property and antibiotics by Mantel’s tests. The black triangle represented Pearson’s correlations coefficients between physicochemical properties. The blue square represented Pearson’s correlations coefficients between physicochemical properties and antibiotics. The red triangle represented Pearson’s Correlations Coefficients between antibiotics. Edge width corresponded to Mantel’s r statistic for the corresponding distance correlations, and edge color denoted the statistical significance based on 9999 permutations.

Supplementary Figure S8 **Environmental drivers of microbial community composition of physicochemical properties and antibiotics in impacted and less-impacted groups for water and sediment samples, respectively** Taxonomic composition and functional composition were related to each physicochemical property and antibiotic by Mantel’s tests. In all figures, edge width corresponded to Mantel’s R statistic for the corresponding distance correlations, and edge color denoted the statistical significance based on 9999 permutations on the right of all figures by gradient descent. The abbreviations of physicochemical properties and antibiotics were seen “**Physicochemical characterization and antibiotic analysis**”. **A.** Pairwise comparisons of environmental factors of impacted group in sediment samples were shown, with a color gradient denoting Pearson’s Correlations Coefficients (PCC). Taxonomic composition and functional composition were related to each environmental factor by Mantel’s tests. **B.** Pairwise comparisons of environmental factors of less-impacted group in sediment samples were shown, with a color gradient denoting Pearson’s correlations coefficients. Taxonomic composition and functional composition were related to each environmental factor by Mantel’s tests. **C.** Pairwise comparisons of environmental factors of impacted group in water samples were shown, with a color gradient denoting Pearson’s correlations coefficients. Taxonomic composition and functional composition were related to each environmental factor by Mantel’s tests. **D.** Pairwise comparisons of environmental factors of less-impacted group in water samples were shown, with a color gradient denoting Pearson’s correlations coefficients. Taxonomic composition and functional composition were related to each environmental factor by Mantel’s tests.

Supplementary Figure S9 **The correlation analysis between the relative abundances of OTUs and the concentrations of physicochemical properties in water samples based on Pearson’s Correlation Coefficient with corrected *P* value** Pearson’s Correlation Coefficient analysis was conducted to explore the linear relationship between OTUs and physicochemical properties, which were transformed to z-scores before calculation, in water samples. TP represents total phosphorus, PO^43−^-P represents orthophosphate, TN represents total nitrogen, NH^4+^-N represents ammonia nitrogen, NO^3−^-N represents nitrate nitrogen, NO^2−^-N represented nitrite nitrogen. Denovo 44775 in **A** and **B** represents k__Bacteria; p__Bacteroidetes; c__Sphingobacteriia; o__Sphingobacteriales; f__Sphingobacteriaceae. Denovo 71301 in **C**, **D**, and **E** represents k__Bacteria; p__Firmicutes; c__Bacilli; o__Bacillales; f__Bacillaceae; g__Bacillus; s__flexus. Denovo 75752 in **F** represents k__Bacteria; p__Proteobacteria; c__Deltaproteobacteria; o__Bdellovibrionales; f__Bdellovibrionaceae; g__Bdellovibrio. Denovo 84762 in G represents k__Bacteria; p__Acidobacteria; c__BPC102; o__MVS-40. H. Denovo 89140 represents k__Bacteria; p__Proteobacteria; c__Betaproteobacteria; o__Rhodocyclales; f__Rhodocyclaceae; g__C39 (Table S10).

Supplementary Figure S10 **The correlation analysis between the relative abundances of OTUs and the concentrations of antibiotics in water samples based on Pearson’s Correlation Coefficient with corrected *P* value** Pearson’s Correlation Coefficient analysis was conducted to explore the linear relationship between OTUs and antibiotics, which were transformed to z-scores before calculation, in water samples. **A.** Denovo 9842 represents k__Bacteria; p__Proteobacteria; c__Betaproteobacteria; o__Burkholderiales; f__Comamonadaceae. CTC represents chlortetracycline. **B.** Denovo 71031 represents k__Bacteria; p__Firmicutes; c__Bacilli; o__Bacillales; f__Bacillaceae; g__Bacillus; s__flexus. OTC represents oxytetracycline (Table S10).

Supplementary Figure S11 **The correlation analysis between the relative abundances of OTUs and the concentrations of physicochemical properties in sediment samples based on Pearson’s Correlation Coefficient with corrected *P* value** Pearson’s Correlation Coefficient analysis was conducted to explore the linear relationship between OTUs and physicochemical properties, which were transformed to z-scores before calculation, in sediment samples. Sed-OM represents the organic matter content in sediment. **A.** Denovo 18861 represents k__Bacteria; p__Bacteroidetes; c__Saprospirae; o__Saprospirales; f__Chitinophagaceae. **B.** Denovo 31743 represents k__Bacteria; p__Firmicutes; c__Clostridia; o__Clostridiales; f__Clostridiaceae; g__Clostridium (Table S10).

Supplementary Figure S12 **The correlation analysis between the relative abundances of OTUs and the concentrations of antibiotics in sediment samples based on Pearson’s Correlation Coefficient with corrected *P* value** Pearson’s Correlation Coefficient analysis was conducted to explore the linear relationship between OTUs and antibiotics, which were transformed to z-scores before calculation, in sediment samples. **A.** Denovo 38876 represents k__Bacteria; p__Chloroflexi; c__Anaerolineae; o__Caldilineales; f__Caldilineaceae; g__Caldilinea. TC represents tetracycline. **B.** Denovo 52303 represents k__Bacteria; p__Proteobacteria; c__Alphaproteobacteria; o__Caulobacterales; f__Caulobacteraceae. SMD represents sulfadimidine. **C.** Denovo 56244 represents k__Bacteria; p__Firmicutes; c__Clostridia; o__Clostridiales; f__Clostridiaceae. CIP represents ciprofloxacin. **D.** Denovo 84756 represents k__Bacteria; p__Firmicutes; c__Bacilli; o__Bacillales; f__Planococcaceae. SMR represented sulfamerazine. E. Denovo 124422 represents k__Bacteria; p__Bacteroidetes; c__Flavobacteriia; o__Flavobacteriales; f__Flavobacteriaceae; g__Flavobacterium (Table S10). SMZ represents sulfamethoxazole.
