# Supplementary material for: Agricultural Risk Factors Influence Microbial Ecology in Honghu Lake
Source: Genomics Proteomics Bioinformatics. 2019 Apr 23;17(1):76–90. doi: 10.1016/j.gpb.2018.04.008 (PMC6520916; doi:10.1016/j.gpb.2018.04.008)
Supplement: Supplementary File S1 — Supplementary information for ‘Agricultural Pollution Risks Influence Microbial Ecology in Honghu Lake’ [file mmc1.doc]

**File S1**

**Agricultural Pollution Risks Influence Microbial Ecology in Honghu Lake**

Maozhen Han^1,2,#,a^, Melissa Dsouza^3,4,5,#,b^, Chunyu Zhou^1,#,c^, Hongjun Li^1,d^, Junqian Zhang^6,e^, Chaoyun Chen^1,f^, Qi Yao^1,g^, Chaofang Zhong^1,h^, Hao Zhou^1,i^, Jack A Gilbert^3,4,5,*,j^, Zhi Wang^2,*,k^, Kang Ning^1,*,l^

*^1^ Key Laboratory of Molecular Biophysics of the Ministry of Education, Hubei Key Laboratory of Bioinformatics and Molecular-imaging, Department of Bioinformatics and Systems Biology, College of Life Science and Technology, Huazhong University of Science and Technology, Wuhan, Hubei 430074, China*

*^2^ Key Laboratory for Environment and Disaster Monitoring and Evaluation of Hubei, Institute of Geodesy and Geophysics, Chinese Academy of Sciences, Wuhan, Hubei 430077, China*

*^3^ The Microbiome Center, Department of Surgery, University of Chicago, Chicago, IL, 60637, USA*

*^4^ Argonne National Laboratory, Biosciences Division, Lemont, IL, 60439, USA*

*^5^* *Marine Biological Laboratory, Woods Hole, MA, 02543, USA*

*^6^ State Key Laboratory of Water Ecology and Biotechnology, Institute of Hydrobiology, Chinese Academy of Sciences, Wuhan, Hubei 430072, China*

^#^ Equal contribution.

^*^ Corresponding authors.

E-mail: [ningkang@hust.edu.cn](mailto:ningkang@hust.edu.cn) (Ning K), [zwang@whigg.ac.cn](mailto:zwang@whigg.ac.cn) (Wang Z), [gilbertjack@gmail.com](mailto:gilbertjack@gmail.com) (Gilbert JA).

Running title: *Han M et al / Microbiota in Honghu Lake*

^a^ ORCID: 0000-0002-5958-1941.

^b^ ORCID: 0000-0001-7969-0857.

^c^ ORCID: 0000-0002-6102-5629.

^d^ ORCID: 0000-0002-8057-6186.

^e^ ORCID: 0000-0001-8141-7622.

^f^ ORCID: 0000-0002-5270-5278.

^g^ ORCID: 0000-0001-5275-0100.

^h^ ORCID: 0000-0002-3509-3628.

^i^ ORCID: 0000-0002-9547-4617.

^j^ ORCID: 0000-0001-7920-7001.

^k^ ORCID: 0000-0002-6832-7013.

^l^ ORCID: 0000-0003-3325-5387.

**Content**

[Materials and Methods 4](#__RefHeading___Toc510432214)

[Physicochemical characterization and antibiotic analysis 4](#__RefHeading___Toc510432215)

[DNA extraction and 16S rRNA gene sequencing 5](#__RefHeading___Toc510432216)

[Results and discussions 7](#__RefHeading___Toc510432217)

[Physicochemical and antibiotic characterization 7](#__RefHeading___Toc510432218)

[General statistics of sequencing results 7](#__RefHeading___Toc510432219)

[Characterization and composition differences of microbial communities 8](#__RefHeading___Toc510432220)

[Core-OTUs and Pan-OTUs of microbial communities 8](#__RefHeading___Toc510432221)

[Analysis of the relationships between physicochemical properties, antibiotics and microbial communities 10](#__RefHeading___Toc510432222)

[References 12](#__RefHeading___Toc510432223)

## Materials and methods

### Physicochemical characterization and antibiotic analysis

*Antibiotics and measuring standards*

13 antibiotics were selected for this study based on their high frequent use in China. These antibiotics could be classiﬁed into three groups, sulfonamides (SAs), including sulfadiazine (SDZ), sulfamerazine (SMR), sulfamater (SFM), sulfadimidine (SMD), sulfamonomethoxine (SMM), sulfamethoxazole (SMZ); fluoroquinolones (FQs), including fleroxacin (FLE), ofloxacin (OFL), ciprofloxacin (CIP), diﬂoxacin (DIF); and tetracyclines group (TCs), including tetracycline (TC), oxytetracycline (OTC) and chlortetracycline (CTC). Standards for SDZ, SMR, SFM, and DIF with purities of > 99% were obtained from Dr Ehrenstorfer (Augsburg, Germany), and the other antibiotic standards, with purities of > 98% were purchased from the National Institute for the Control of Pharmaceutical and Biological Products (Beijing, China). Individual antibiotic standard stock solutions with concentrations up to 1,000 mg/L were prepared in 50% methanol and stored in the dark at −20^o^C before use. Mixed working solutions with different concentrations were prepared by diluting the stock solutions before each analytical process.

*Antibiotics extraction process*

Extraction procedures for the 13 antibiotics in water samples were optimized according to Tong et al. , with a few modiﬁcations. Briefly, 1500 ml water samples were filtered through with a muffle furnace- burned glass filter (Ф 47 mm, pore size 0.45 μm). The pH value was adjusted to 3 with HCl (6 M). Then, 0.8 g Na_2_EDTA was added to the filtrate to complex divalent cations. Solid phase extraction (SPE) was conducted using the Supelco Visiprep SPE system (Supelco) and oasis hydrophilic–lipophilic balance (HLB) cartridges (6 mL/500 mg, Waters, UK) were used to gather antibiotics. The cartridges were pre-treated with 5 mL methanol followed by 5 mL ultrapure water and 5 mL ultrapure water (pH = 3), after which samples were passed through at a loading rate of 2–3 mL/min. After all samples were loaded, the HLB cartridges were washed with 10 mL ultrapure water and 10 mL 5% methanol. Then, the HLB cartridge was eluted with 5 mL methanol and 5 mL 2% ammonia methanol. The ﬁnal eluate was collected into a glass tube and evaporated to dryness using a rotary evaporator (Yarong, China), re-suspended in 0.6 mL of 50% methanol and stored at −20^o^C for further analysis.

For sediment sampling, according to the analytical procedures of Li et al*.* , the analytical procedure with a tiny optimization was used in this study. Briefly, 1.5 g of each freeze-dried and powdered sediment sample was weighted into a 50 mL glass tube, followed by addition of 10 mL extraction [0.1 M citrate buffer solution (pH 3) containing 1 mM Na_2_EDTA: Methanol = 1:1 (v/v)]. The sample was vortexed for 1 min and treated ultrasonically for 15 min, followed by centrifugation. This extraction process was repeated for three times. The extract was combined into a round-bottom ﬂask, concentrated with a rotary evaporator at 50^o^C to remove the organic solvent, and diluted to 200 mL with ultrapure water to make sure the organic solvent in solution had a concentration of less than 5%. The following extraction process was the same as water sample.

***Antibiotics analytical method****:* Instrumental determinations of antibiotics were performed by a 2695 Waters Alliance system (Milford, MA) equipped with an autosampler-controlled binary gradient system, a micro vacuum degasser and a 2998 Photodiode Array (PDA) detector. Separations were obtained using Waters XTerra C18 column (4.6 ×150 mm, particle size 5 μm) at a ﬂow rate of 0.8 mL/min. Chromatographic and detector operating conditions are referred to Hu et al. , Zhao et al. and Summa et al. , and optimized. Ten mixed standards for SAs and FQs, and three mixed of TCs were analyzed by the HPLC operating conditions to form the calibration. Line curve ﬁts (Each antibiotic concentration ranged from 0.1−5 mg/L) were used for all analyses and correlation coefﬁcients were higher than 0.995. Qualitative analysis of antibiotics was based on the comparison of the retention time and 3-dimensional (3D) fingerprint between unknown substances and standard products . The quantitative analysis was according to the standard curve of each antibiotic.

Among the 13 kinds of antibiotics we measured, 9 kinds of antibiotics, including TC, OTC, CTC, SDZ, SMR, SMD, OFL, CIP, and SMZ, were selected for analysis in this study.

### DNA extraction and 16S rRNA gene sequencing

***DNA extraction:*** DNA was extracted from all filter and sediment samples using a modified hexadecyltrimethylammonium bromide (CTAB) method . Water filter membranes and 0.5 g of dried sediment were used for DNA extraction, respectively. Briefly, water filter membranes were cut into quarters and then utilized for DNA extraction. To extract DNA from sediment samples, 0.5 g of dried sediment was utilized. First, filter membranes or sediment samples were dissolved in a 1 ml solution of 0.1 M Tris-HCl, 20 mM EDTA (pH 8.0) with 0.1−0.2 μm diameter glass beads. Then, 2 ml of a lysis solution comprising 2% CTAB, 0.1 M Tris-HC1 (pH 8.0), 20 mM EDTA (pH 8.0), 1.4 M NaCl, and 100 μL 10% SDS, 10 μL 10 mg/mL Proteinase K (Sigma, MO), and 100 μL β-Mercaptoethanol (Amresco, OH) were added to the tube and incubated at 65^o^C for 1 h with occasional stirring. Subsequently, 0.5 ml of phenol:chloroform:isoamyl alcohol (25:24:1) was added, and the entire reaction was centrifuged at 16,000 g for 15 min. The aqueous phase was spun again with 0.5 ml of phenol:chloroform:isoamyl alcohol (25:24:1). The aqueous phase was then transferred to a clean microcentrifuge tube containing 0.5 ml chloroform:isoamyl alcohol (24:1) and the reaction was centrifuged at 16,000 g for 15 min. To precipitate DNA, the supernatant was incubated at -20^o^C with 0.6 vol cold isopropanol or 30 min. The precipitated DNA was washed with 75% ethanol, dissolved in TE buffer and stored at −20^o^C before use.

## Results and discussions

### Physicochemical and antibiotic characterization

For the physicochemical properties, we have measured samples from water and sediment, where there were differences among ORP and pH (Table S1). These differences included: firstly, the oxidizing reactions were more easily carried in sediment samples than water samples for the reason that the ORP values of sediment samples were negative value and those of water samples were positive value; secondly, the pH values of water and sediment samples showed that the alkalinity of water samples (8.69−9.55) were higher than sediment samples (6.41−7.66, Table S1 and Table S2). Meanwhile, for samples from different types of sampling sites (lake water or sediment, pond water or sediment, river water or sediment), there were also differences among different physicochemical properties. Briefly, the concentration of TN and TP of lake water were lower than those of pond water and river water (Table S1) and the concentration of Sed-OM, Sed-NH_4_^+^-N, Sed-LP, Sed-TN and Sed-TP of almost all lake sediment samples were higher than those of pond sediment and river sediment (Table S2).

### General statistics of sequencing results

In this study, a total of 4,441,405 paired-end raw reads were extracted from the 28 samples. 1,746,325 and 2,695,080 paired-end raw reads were obtained in 14 water samples and 14 sediment samples, respectively. The number of reads per sample ranged from 91,819 to 275,314, with an average of 158,621 (Table S4). After a series of quality control (QC) methods, 3,715,787 high quality reads (average 132,706 reads per sample) were obtained for downstream analysis. We set the threshold to 0.001% to remove the low-abundance OTUs. Finally, a total of 7785 OTUs were obtained from 3,258,498 reads of 28 samples, ranging from 2486 to 5055 OTUs per water sample and from 3473 to 5015 per sediment sample. Although the maximum OTUs in P2W sample, the average OTUs of water samples (3270) was lower than that of sediment samples (4219, Table S4). Although the rarefaction curves based on the observed-OTUs have shown that all the samples didn’t approach the saturation plateau, the Shannon richness have shown that all samples have approached the saturation plateau suggesting that we had captured most bacteria of the community.

### Characterization and composition differences of microbial communities

Based on the classified information and the taxonomy information of OTUs, a total of 53 phyla were found and 9 of them were identified as major phyla (average abundance > 1%). Among these phyla, the top 13 phyla (sorted by the average relative abundance for all samples) were chosen and illustrated in Figure 2A. *Proteobacteria* was the most abundant phylum in all water samples and sediment samples, accounting for 39.96% ± 11.19% in community (average relative abundance per sample, Figure 2A). According to the statistical results of taxonomical structure at phylum level, there were huge differences among water samples and sediment samples, for example, the other dominant phyla were *Actinobacteria* (18.51%±7.33%), *Bacteroidetes* (20.66%±5.47%), *Cyanobacteria* (10.44%±6.36%), *Planctomycetes* (4.65%±1.60%) and *Verrucomicrobia* (2.47%±1.03%) in water samples, while the other dominant phyla were *Firmicutes* (25.27%±12.44%), *Chloroflexi* (8.91%±4.14%) and *Acidobacteria* (2.73%±1.41%) in sediment samples (Figure 2A and Figure 2B). In summary, among the rest of 12 phyla, the relative abundance of these phyla all showed significant difference in water samples and sediment samples (*t*-test, all *P* < 0.01, Figure 2B), except *Chlorobi* (0.91%±0.66%, *t*-test, *P* > 0.05, Figure 2B).

Additionally, analysis results at genus level showed that an unclassified genus of *ACK-M1* family (13.41%±6.61%) was the most abundant genus in water samples. Moreover, *Acinetobacter* genus (22.06%±18.1%) was identified as the most abundant genus in sediment samples. In addition, the abundance of *Acinetobacter* genus was considerably various (ranging from 1.51% to 62.58%, the maximum value in L1S sample).

### Core-OTUs and pan-OTUs of microbial communities

The results revealed that 7785, 6578 and 6736 pan-OTUs and 58, 530 and 849 core-OTUs existed in all samples, water samples and sediment samples (Figure 3), respectively. More specifically, the 58 core-OTUs, accounting for 7.99%±4.53% in all samples and these OTUs can be classified into 8 phyla (Table S5), which manifested diverse biological properties. The dominant core-OTUs appeared as *Protebacteria*, whose number was 35, accounting for 4.80%±2.90%. Furthermore, *Pseudomonas* genus was detected as the most abundant genus in the core-OTUs (1.27%±1.78%, Table S5), which was associated as the organic metabolism , biofilm formation , production of secondary metabolites and antibiotic resistance . For other OTUs, we investigated that some were tightly associated with the utilization of nitrogen, such as denovo16545, denovo23072, denovo34896, denovo78136, denovo87422, denovo92921, denovo122070, belonging to *Rhizobiales* (an order of *Protebacteria*), denovo121222 (Table S5), belonging to *Oxalobacteraceae* (a family of *Protebacteria*), which fix nitrogen as their sole energy source, and denovo46517, denovo53046 and denovo97814, belonging to *Synechococcus* (a genus of *Cyanobacteria*), which tend to be much more abundant in nutrient rich environments. Besides, other energy metabolism abilities were detected as photosynthesis of denovo13272 (in *Rhodobacter* of *Proteobacteria*), denovo108418 (in *Rhodospirillales* of *Proteobacteria*), the use of organics as energy and carbon sources of denovo1411, denovo23148, denovo24094 (nylon-eating and in *Flavobacterium* genus of *Proteobacteria*), denovo17757 (degrading aromatic compounds and in *Novosphingobium* genus of *Proteobacteria*) and denovo60018 (a *methanotroph* and in *Methylococcaceae* family of *Proteobacteria*), and reduction of sulfate of denovo7309 (in *Desulfobacteraceae* family of *Proteobacteria*) and denovo124556 (in *Syntrophaceae* family of *Proteobacteria*). Besides, we also found out some OTUs related with production of antibiotics in considerable abundance, including denovo130330, denovo76394, denovo76881 and denovo24563 in *Acidobacteria* phylum, denovo83113 and denovo90890 (Table S5) in *Proteobacteria* phylum, whose co-occurrence within water and sediment may illustrate the active behavior of these bacteria over a long period of time in the lake ecosystem and the condition of antibiotics in water body to a certain extent. As to the core-OTUs of water samples and sediment samples, the results showed that the core-OTUs would be sought out with the increasing of number of samples. Based on analysis results, the core-OTUs of water samples were accounting for 61.21%±7.43%, while those of sediment samples were 67.94%±10.45%. The average relative abundance of 10 different taxa in water samples were more than 1% and the most abundant OTUs belonged to *Pelagibacteraceae* family, accounting for 3.80%, related species of which were scavengers and fed on dissolved organic carbon and nitrogen and with the acquirement for reduced sulfur . Meanwhile, there were 10 different taxa existed in sediment samples, and the average relative abundance was also more than 1%. However, the differences were mainly associated with the different genus. Among the top 10 core-OTUs, the dominant genus (average relative abundance > 2%) were *Actinobacteria* (10.13%±10.03%), *Paenisporosarcina* (8.68%±8.30%) and *Clostridium* (2.81%±1.66%).

### Analysis of the relationships between physicochemical properties, antibiotics and microbial communities

To explore correlations between OTUs and environmental variables (including physicochemical properties and antibiotics data), their Pearson Correlation Coeffcients were calculated for water samples and sediment samples, respectively. The results of this analysis showed that there were linear relationships between OTU relative abundances and environmental factors (Figure S9 **-** Figure S12). The relative abundances of water microbial OTUs including denovo 71031 (*Bacillus flexus*), denovo 75752 (*Bdellovibrio* genus), and denovo 84762 (*MVS-40* order) (Table S10) were correlated with TN (Figure S9C, F, and G). Likewise, the relative abundances water OTUs including denovo 44775 (*Sphingobacteriaceae* family) and devono 71031 (*Bacillus flexus*) were correlated with NH_4_^+^-N (Figure S9A and D) and OTUs denovo 44775 (*Sphingobacteriaceae* family), denovo 89140 (*Rhodocyclaceae* family), and denovo 71031 (*Bacillus flexus*) (Table S10) were correlated with NO_3_^-^-N, NO_2_^-^-N, and PO_4_^3-^-P (Figure S9 B, H and E), respectively. Using antibiotic data, the relative abundances of OTUs including denovo 9842 (*Comamonadaceae* family) and denovo 71031 (*Bacillus flexus*) (Table S10) were were correlated with CTC and OTC (Figure S10**)**, respectively.

Using physicochemical data from sediment samples, the relative abundances of OTUs including denovo 18861 (*Chitinophagaceae* family) and denovo 31743 (*Clostridium* genus) (Table S10) were correlated with Sed-OM (Figure S11**)**. Likewise using antibiotic data, the relative abundance of OTU denovo 52303 (*Caulobacteraceae* family) was correlated with SMD, denovo 56244 (*Clostridiaceae* family) with CZP, denovo 84756 (*Planococcaceae* family) with SMR, denovo 124422 (*Flavobacterium* genus) with SMZ (Table S10), and denovo 38876 with TC (Figure S12**)**. Consistent with observations from a previous study, OTU denovo 38876 was classified as a member of *Caldilinea* (class *Anaerolineae*) – a known tetracycline resistant bacteria.

## References

[1] Tong L, Huang S, Wang Y, Liu H, Li M. Occurrence of antibiotics in the aquatic environment of Jianghan Plain, central China. Sci Total Environ 2014;497−498:180−7.

[2] Li C, Chen J, Wang J, Ma Z, Han P, Luan Y, et al. Occurrence of antibiotics in soils and manures from greenhouse vegetable production bases of Beijing, China and an associated risk assessment. Sci Total Environ 2015;521−522:101−7.

[3] Hu XG, Yi L, Zhou QX, Xu L. Determination of thirteen antibiotics residues in manure by solid phase extraction and high performance liquid chromatography. Chinese J Anal Chem 2008;36:1162−6.

[4] Zhao L, Dong YH, Wang H. Residues of veterinary antibiotics in manures from feedlot livestock in eight provinces of China. Sci Total Environ 2010;408:1069−75.

[5] Summa S, Magro SL, Armentano A, Muscarella M. Development and validation of an HPLC/DAD method for the determination of 13 sulphonamides in eggs. Food Chem 2015;187:477−84.

[6] Cheng X, Chen X, Su X, Zhao H, Han M, Bo C, et al. DNA extraction protocol for biological ingredient analysis of LiuWei DiHuang Wan. Genomics Proteomics Bioinformatics 2014;12:137−43.

[7] Atkinson BW, Mudaly DD, Bux F. Contribution of Pseudomonas spp. to phosphorus uptake in the anoxic zone of an anaerobic-anoxic-aerobic continuous activated sludge system. Water Sci Technol 2001;43:139−46.

[8] Hassett DJ, Cuppoletti J, Trapnell B, Lymar SV, Rowe JJ, Yoon SS, et al. Anaerobic metabolism and quorum sensing by Pseudomonas aeruginosa biofilms in chronically infected cystic fibrosis airways: rethinking antibiotic treatment strategies and drug targets. Adv Drug Deliv Rev 2002;54:1425−43.

[9] Ray CG, Ryan KJ. Sherris Medical Microbiology. 4th ed. McGraw Hill; 2004.

[10] Thrash JC, Boyd A, Huggett MJ, Grote J, Carini P, Yoder RJ, et al. Phylogenomic evidence for a common ancestor of mitochondria and the SAR11 clade. Sci Rep 2011;1:13.

[11] Tripp HJ, Kitner JB, Schwalbach MS, Dacey JW, Wilhelm LJ, Giovannoni SJ. SAR11 marine bacteria require exogenous reduced sulphur for growth. Nature 2008;452:741−4.

[12] Huang K, Tang J, Zhang XX, Xu K, Ren H. A comprehensive insight into tetracycline resistant bacteria and antibiotic resistance genes in activated sludge using next-generation sequencing. Int J Mol Sci 2014;15:10083−100.
