# Supplementary figures and images for: Agricultural Risk Factors Influence Microbial Ecology in Honghu Lake
Source: Genomics Proteomics Bioinformatics. 2019 Apr 23;17(1):76–90. doi: 10.1016/j.gpb.2018.04.008 (PMC6520916; doi:10.1016/j.gpb.2018.04.008)

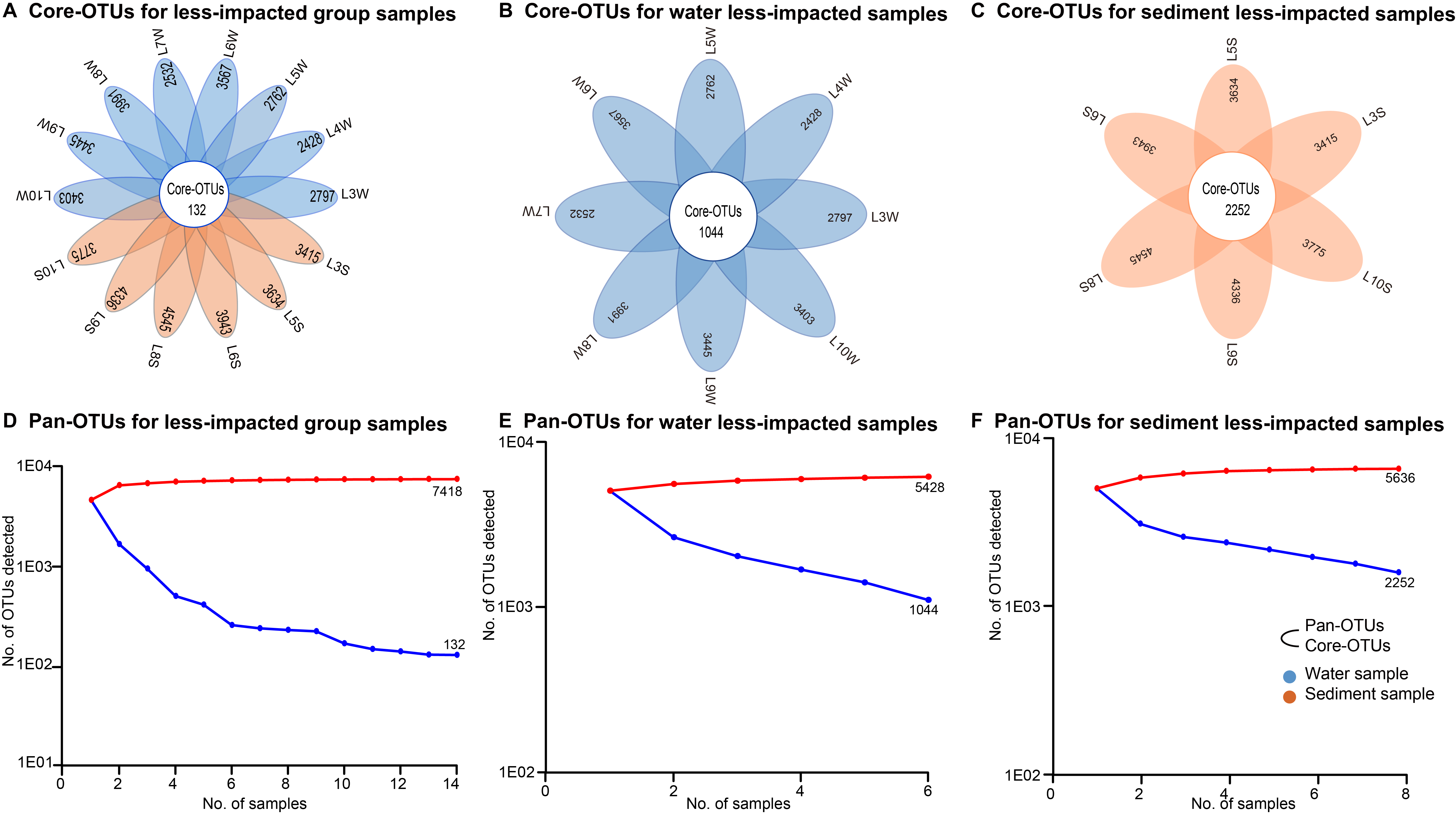

Supplement: Supplementary Figure S1 — The core-OTUs and pan-OTUs of water samples and sediment samples from less-impacted group in Honghu lake The flower plots illustrate the number of shared and specific OTUs. A. Flower plot showing numbers of specific OTUs (in the petals) of each water sample and sediment sample and core-OTUs (in the center) of less-impacted group samples. B. Flower plot showing numbers of specific OTUs (in the petal) and core-OTUs (in the center) of each water sample in less-impacted group. C. Flower plot showing numbers of specific OTUs (in the petal) and core-OTUs (in the center) of each sediment less-impacted group sample. D. OTU accumulation curves for pan-OTUs (upper) and core-OTUs (lower) for less-impacted group samples from Honghu lake. E. OTU accumulation curves for pan-OTUs (upper) and core-OTUs (lower) for all water samples from less-impacted group in Honghu lake. F. OTU accumulation curves for pan-OTUs (upper) and core-OTUs (lower) for all sediment samples from less-impacted group in Honghu lake. [file mmc2.zip › Figure S1 041119.png]

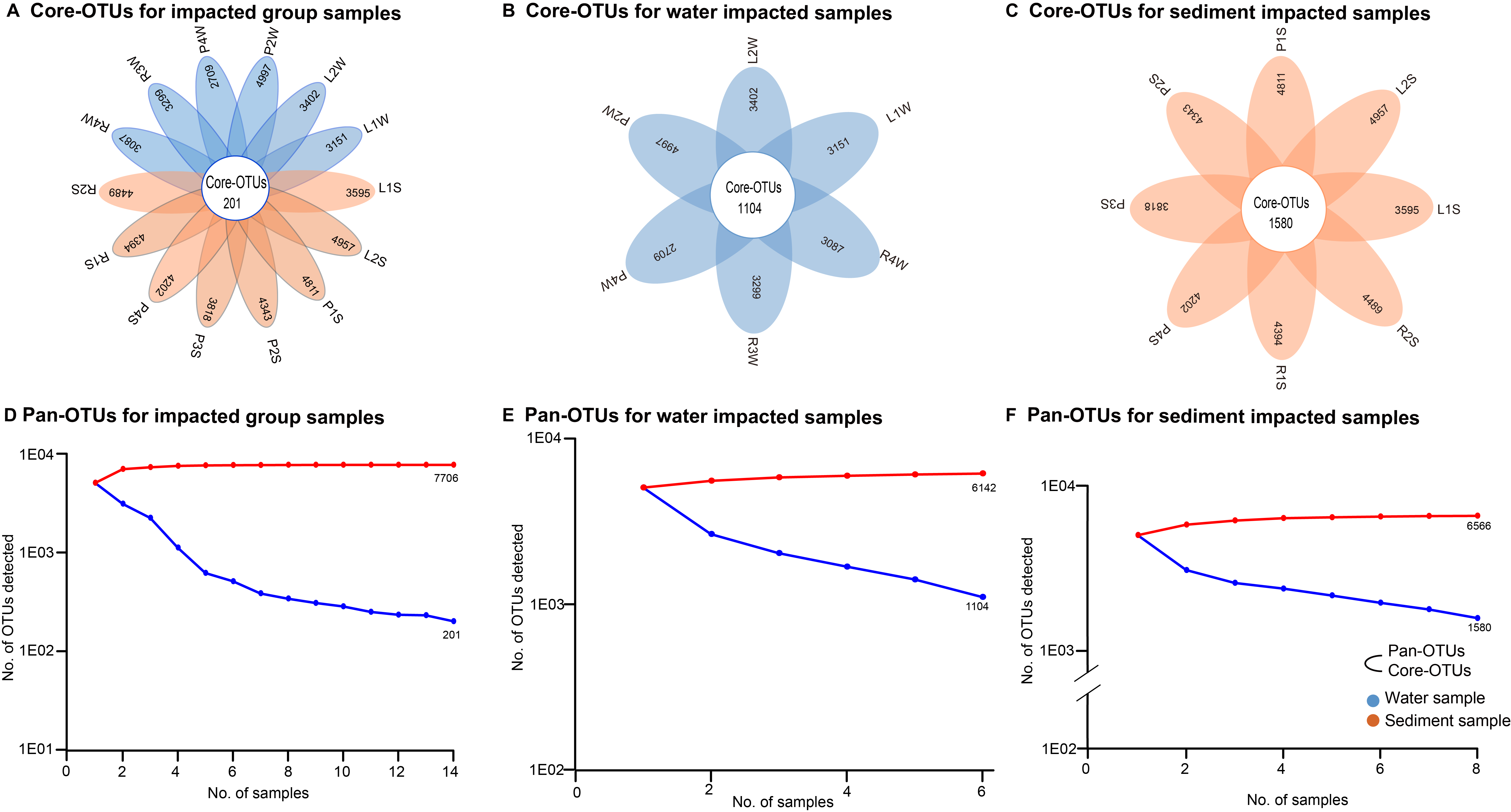

Supplement: Supplementary Figure S2 — The core-OTUs and pan-OTUs of water samples and sediment samples from impacted group in Honghu lake The flower plots illustrate the number of shared and specific OTUs. A. Flower plot showing numbers of specific OTUs (in the petals) of each water sample and sediment sample and core-OTUs (in the center) of impacted group samples. B. Flower plot showing numbers of specific OTUs (in the petal) and core-OTUs (in the center) of each water sample in impacted group. C. Flower plot showing numbers of specific OTUs (in the petal) and core-OTUs (in the center) of each sediment impacted group sample. D. OTU accumulation curves for pan-OTUs (upper) and core-OTUs (lower) for impacted group samples from Honghu lake. E. OTU accumulation curves for pan-OTUs (upper) and core-OTUs (lower) for all water samples from impacted group in Honghu lake. F. OTU accumulation curves for pan-OTUs (upper) and core-OTUs (lower) for all sediment samples from impacted group in Honghu lake. [file mmc3.zip › Figure S2 041119.png]

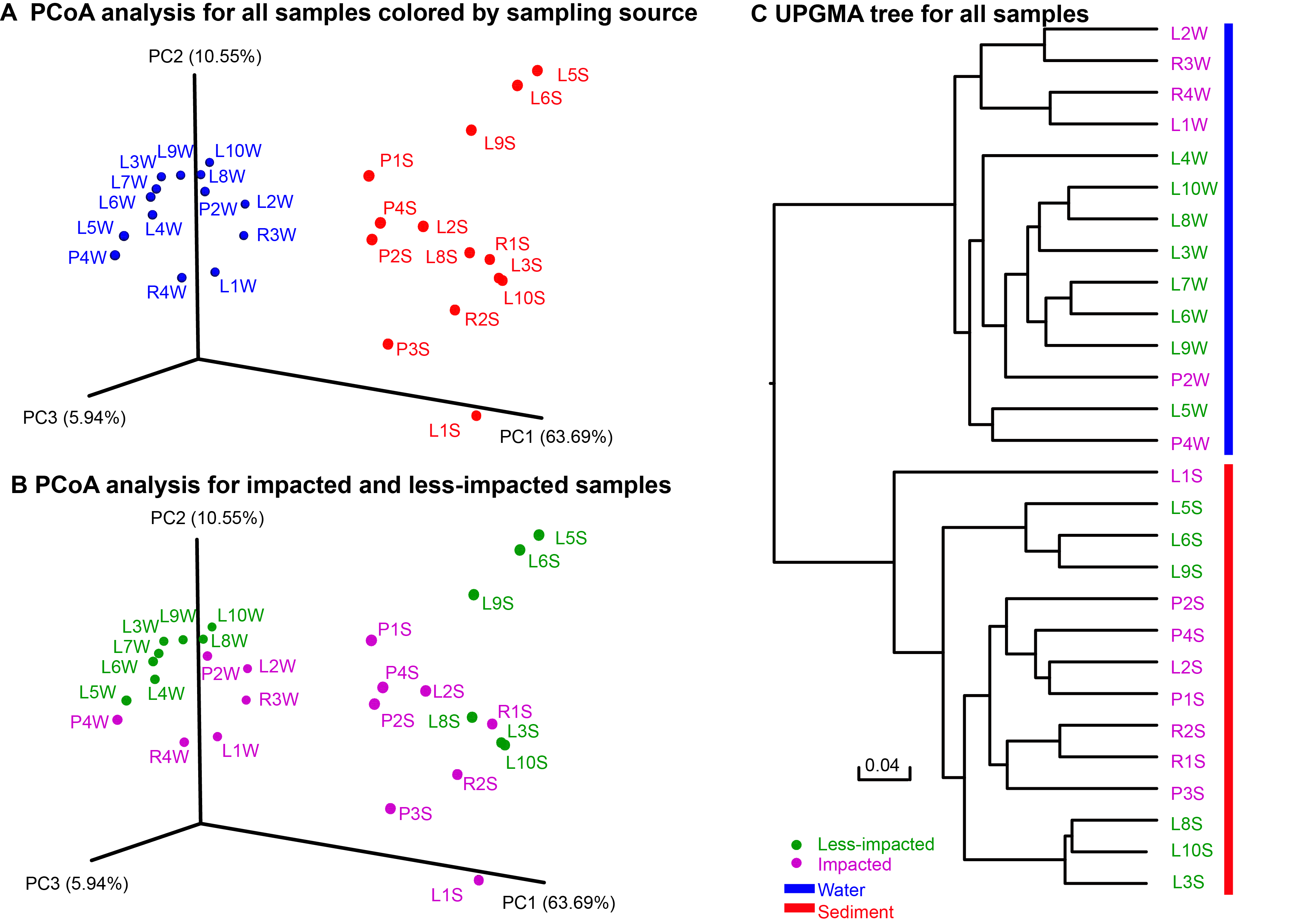

Supplement: Supplementary Figure S3 — PCoA and UPGMA among microbial community taxonomical structure A. PCoA result based on OTU relative abundances of samples using weighted Unifrac metric. The red color and blue color represented the water samples and the sediment samples, respectively. B. PCoA result based on OTU relative abundances of samples using weighted Unifrac metric. The different colors represented different types of samples. C. UPGMA result based on the weighted Unfirac metric used in PCoA. The green and pink circles represent less-impacted group and impacted group, respectively, as well as the blue and red boxes represent water and sediment samples, respectively. The clustering result helps to determine the similarity of the microbial communities between different samples. Consequently, the results showed the microbial communities was correlated with agricultural activities. [file mmc4.zip › Figure S3 041019.png]

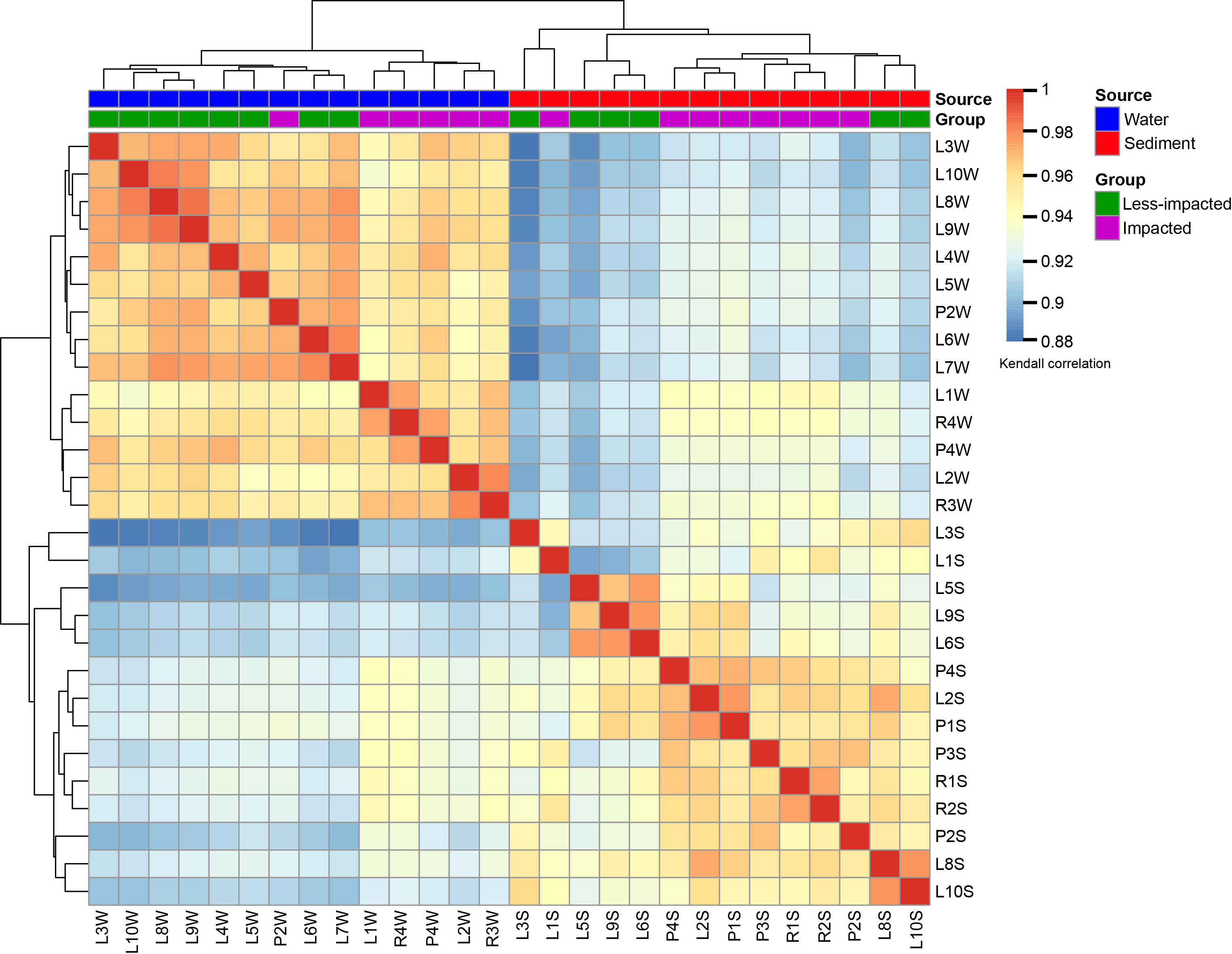

Supplement: Supplementary Figure S4 — Heatmap plot showing the Kendall correlations between water and sediment samples clustered by UPGMA linkage hierarchical clustering [file mmc5.zip › Figure S4 041019.png]

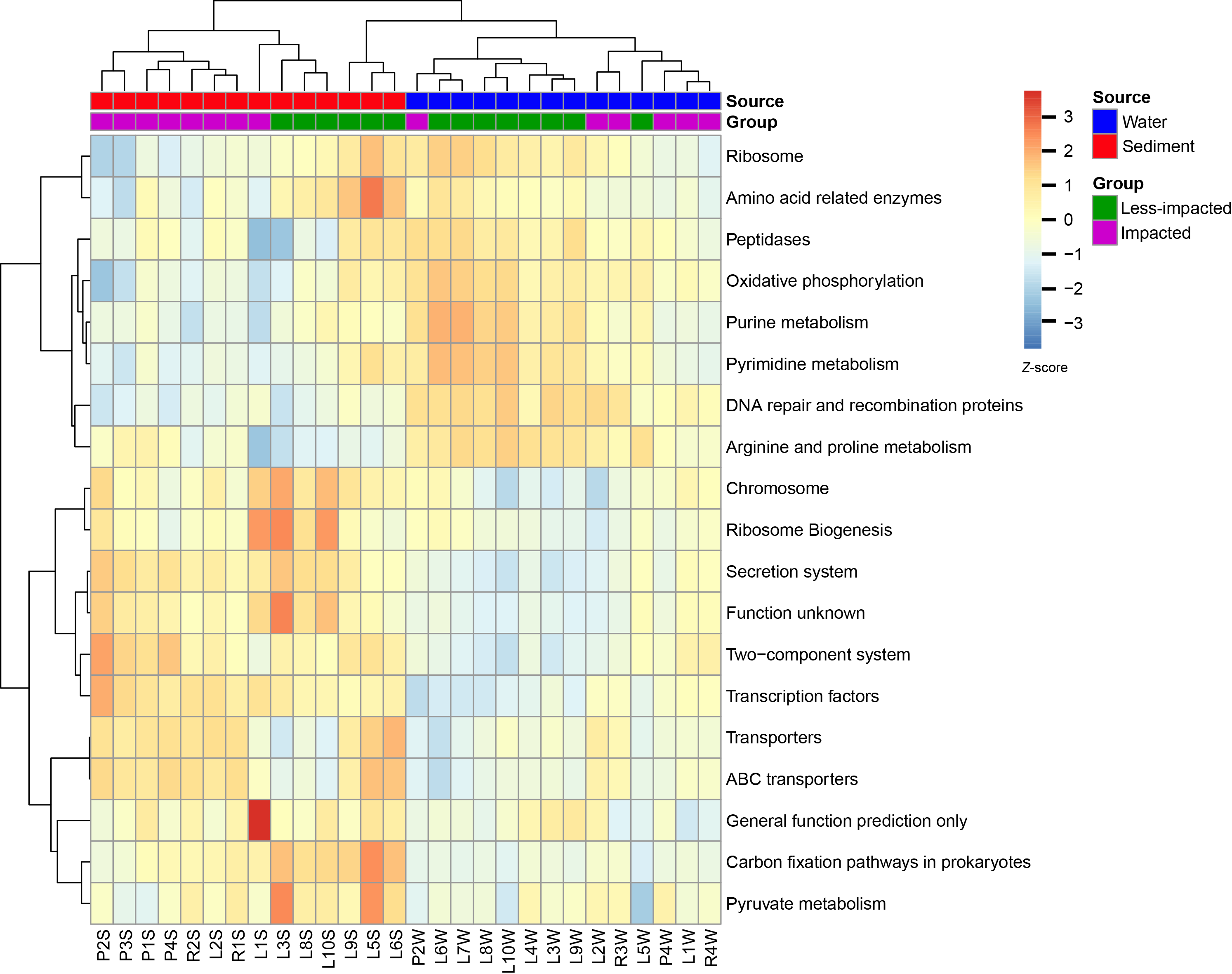

Supplement: Supplementary Figure S5 — Heatmap plot showing the main functional properties between water and sediment samples clustered by Spearman’s correlation coefficient and UPGMA linkage hierarchical clustering The functional categories with relative abundances more than 0.01 in each sample were selected as main functional properties and shown in this figure. [file mmc6.zip › Figure S5 041019.png]

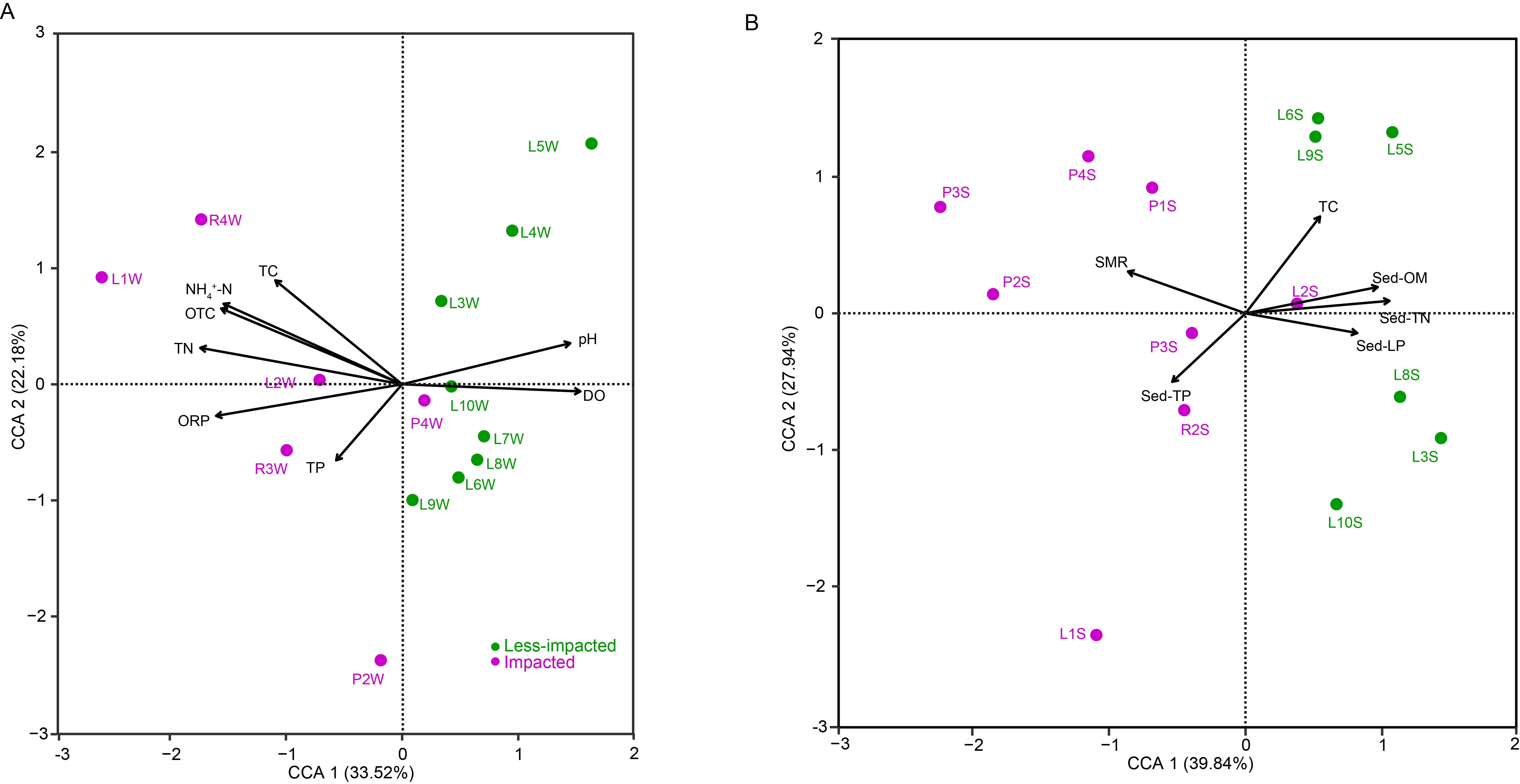

Supplement: Supplementary Figure S6 — Canonical correspondence analysis plots of environmental parameters driving water and sediment microbial community structure Environmental parameters of water samples (A) and sediment samples (B) from Honghu lake. [file mmc7.zip › Figure S6 041019.png]

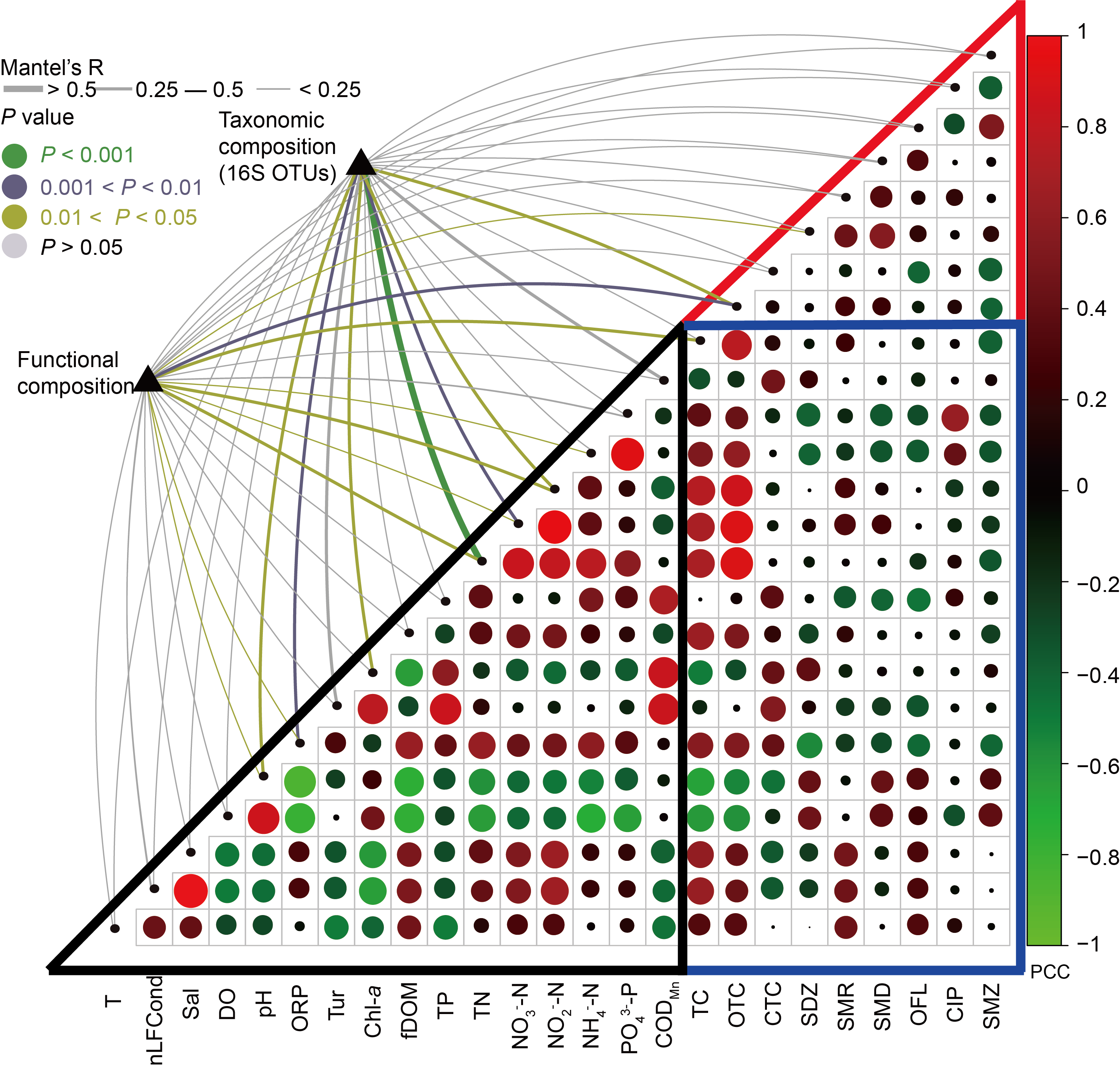

Supplement: Supplementary Figure S7 — Environmental drivers of microbial community composition of water samples Pairwise comparisons of physicochemical properties and antibiotics of water samples were shown, with a color gradient denoting Pearson’s Correlations Coefficients (PCC). Taxonomic composition and functional composition were related to each physicochemical property and antibiotics by Mantel’s tests. The black triangle represented Pearson’s correlations coefficients between physicochemical properties. The blue square represented Pearson’s correlations coefficients between physicochemical properties and antibiotics. The red triangle represented Pearson’s Correlations Coefficients between antibiotics. Edge width corresponded to Mantel’s r statistic for the corresponding distance correlations, and edge color denoted the statistical significance based on 9999 permutations. [file mmc8.zip › Figure S7 041019.png]

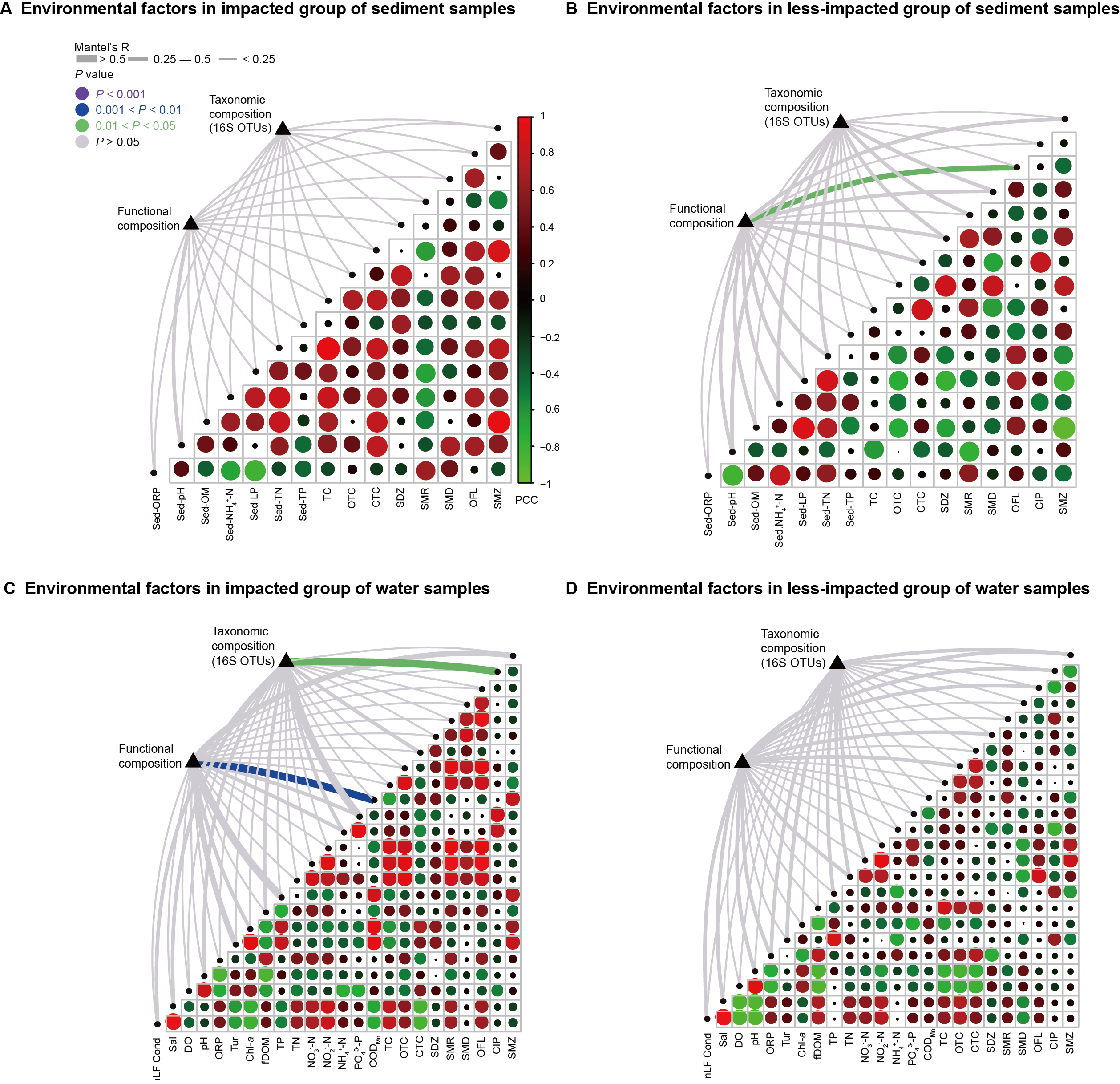

Supplement: Supplementary Figure S8 — Environmental drivers of microbial community composition of physicochemical properties and antibiotics in impacted and less-impacted groups for water and sediment samples, respectively Taxonomic composition and functional composition were related to each physicochemical property and antibiotic by Mantel’s tests. In all figures, edge width corresponded to Mantel’s R statistic for the corresponding distance correlations, and edge color denoted the statistical significance based on 9999 permutations on the right of all figures by gradient descent. The abbreviations of physicochemical properties and antibiotics were seen “Physicochemical characterization and antibiotic analysis”. A. Pairwise comparisons of environmental factors of impacted group in sediment samples were shown, with a color gradient denoting Pearson’s Correlations Coefficients (PCC). Taxonomic composition and functional composition were related to each environmental factor by Mantel’s tests. B. Pairwise comparisons of environmental factors of less-impacted group in sediment samples were shown, with a color gradient denoting Pearson’s correlations coefficients. Taxonomic composition and functional composition were related to each environmental factor by Mantel’s tests. C. Pairwise comparisons of environmental factors of impacted group in water samples were shown, with a color gradient denoting Pearson’s correlations coefficients. Taxonomic composition and functional composition were related to each environmental factor by Mantel’s tests. D. Pairwise comparisons of environmental factors of less-impacted group in water samples were shown, with a color gradient denoting Pearson’s correlations coefficients. Taxonomic composition and functional composition were related to each environmental factor by Mantel’s tests. [file mmc9.zip › Figure S8 041019.png]

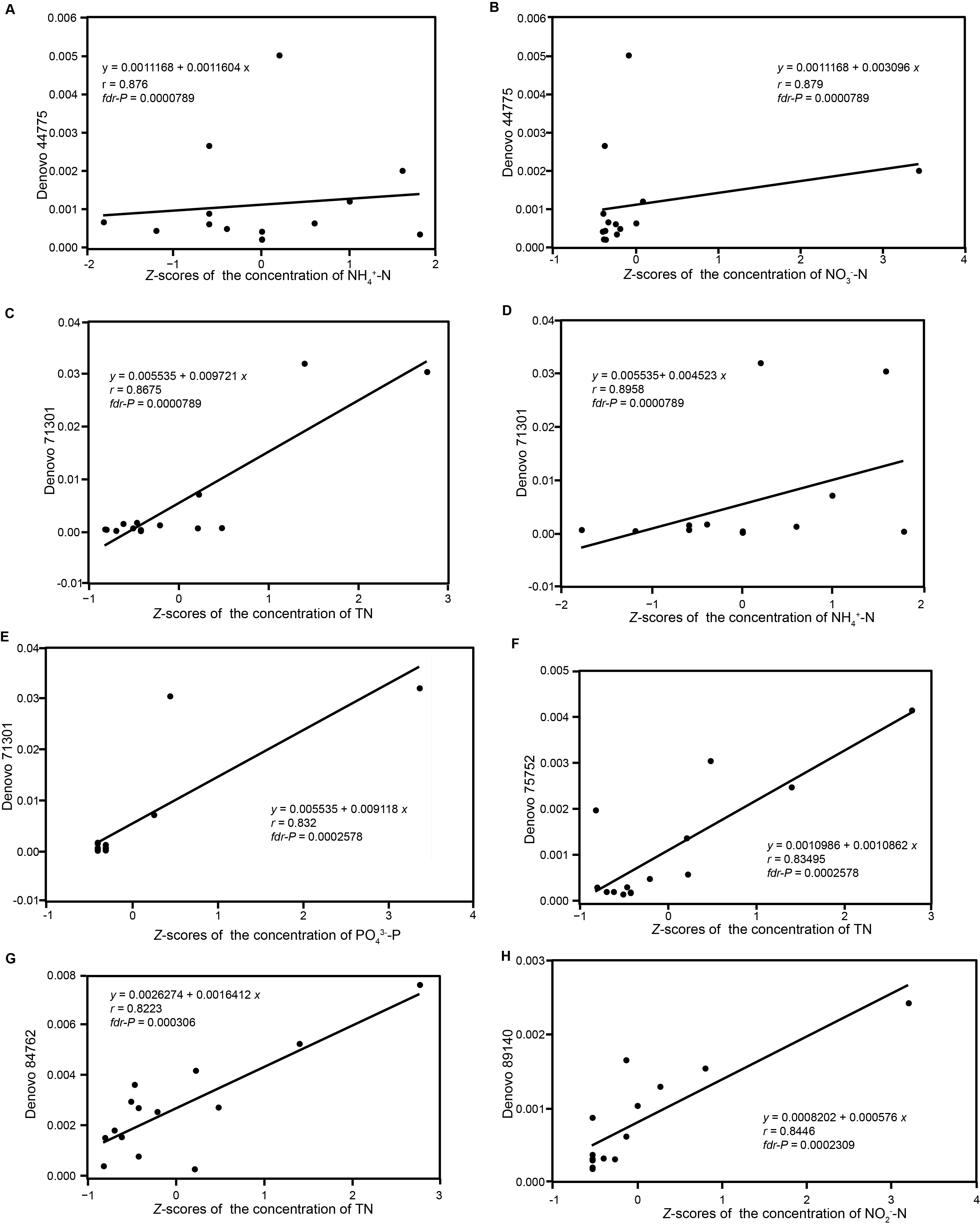

Supplement: Supplementary Figure S9 — The correlation analysis between the relative abundances of OTUs and the concentrations of physicochemical properties in water samples based on Pearson’s Correlation Coefficient with corrected P value Pearson’s Correlation Coefficient analysis was conducted to explore the linear relationship between OTUs and physicochemical properties, which were transformed to z-scores before calculation, in water samples. TP represents total phosphorus, PO43−-P represents orthophosphate, TN represents total nitrogen, NH4+-N represents ammonia nitrogen, NO3−-N represents nitrate nitrogen, NO2−-N represented nitrite nitrogen. Denovo 44775 in A and B represents k__Bacteria; p__Bacteroidetes; c__Sphingobacteriia; o__Sphingobacteriales; f__Sphingobacteriaceae. Denovo 71301 in C, D, and E represents k__Bacteria; p__Firmicutes; c__Bacilli; o__Bacillales; f__Bacillaceae; g__Bacillus; s__flexus. Denovo 75752 in F represents k__Bacteria; p__Proteobacteria; c__Deltaproteobacteria; o__Bdellovibrionales; f__Bdellovibrionaceae; g__Bdellovibrio. Denovo 84762 in G represents k__Bacteria; p__Acidobacteria; c__BPC102; o__MVS-40. H. Denovo 89140 represents k__Bacteria; p__Proteobacteria; c__Betaproteobacteria; o__Rhodocyclales; f__Rhodocyclaceae; g__C39 (Table S10). [file mmc10.zip › Figure S9 041119.png]

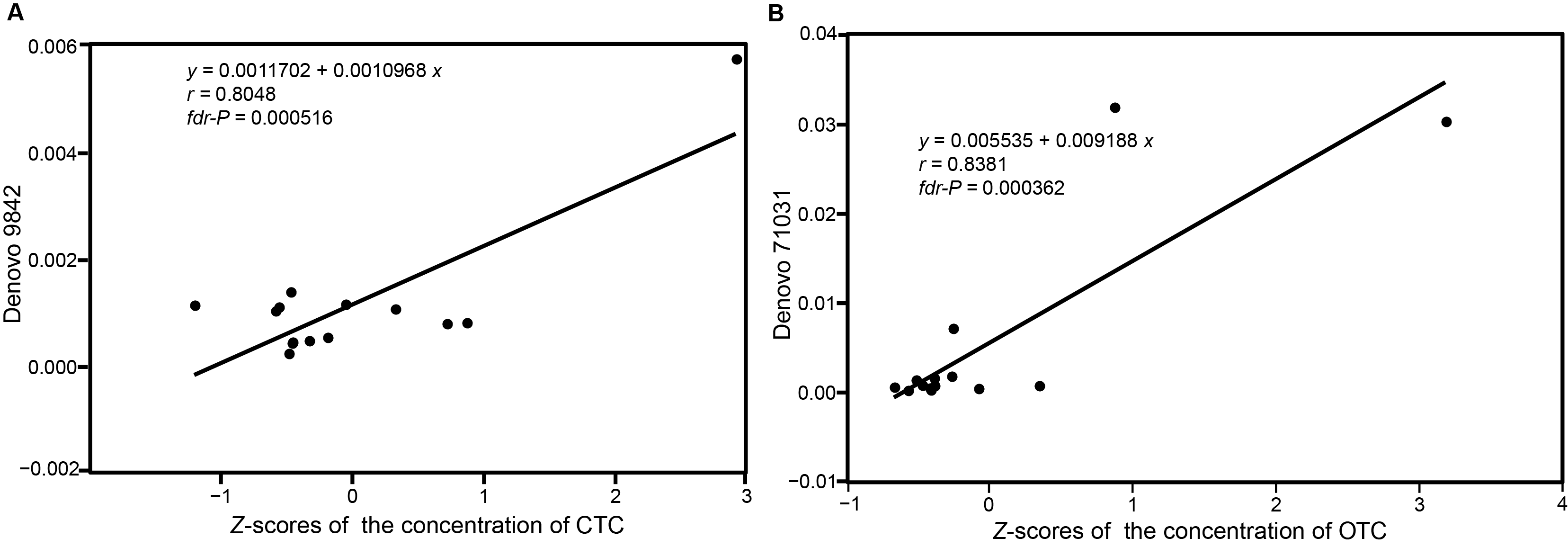

Supplement: Supplementary Figure S10 — The correlation analysis between the relative abundances of OTUs and the concentrations of antibiotics in water samples based on Pearson’s Correlation Coefficient with corrected P value Pearson’s Correlation Coefficient analysis was conducted to explore the linear relationship between OTUs and antibiotics, which were transformed to z-scores before calculation, in water samples. A. Denovo 9842 represents k__Bacteria; p__Proteobacteria; c__Betaproteobacteria; o__Burkholderiales; f__Comamonadaceae. CTC represents chlortetracycline. B. Denovo 71031 represents k__Bacteria; p__Firmicutes; c__Bacilli; o__Bacillales; f__Bacillaceae; g__Bacillus; s__flexus. OTC represents oxytetracycline (Table S10). [file mmc11.zip › Figure S10 041019_modified_201904001632762300.png]

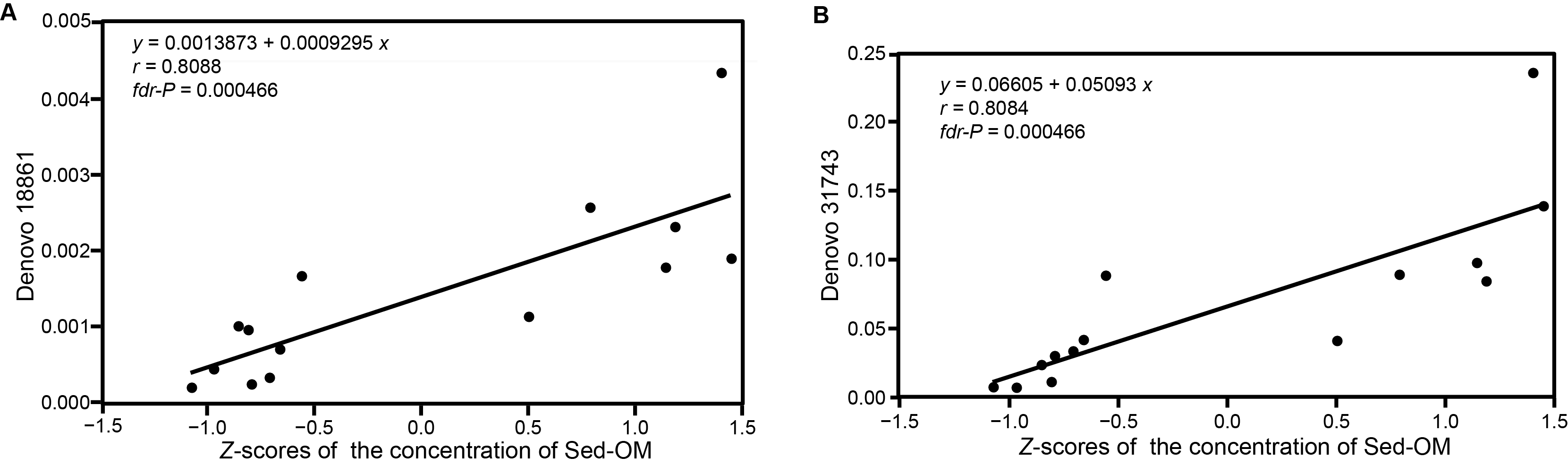

Supplement: Supplementary Figure S11 — The correlation analysis between the relative abundances of OTUs and the concentrations of physicochemical properties in sediment samples based on Pearson’s Correlation Coefficient with corrected P value Pearson’s Correlation Coefficient analysis was conducted to explore the linear relationship between OTUs and physicochemical properties, which were transformed to z-scores before calculation, in sediment samples. Sed-OM represents the organic matter content in sediment. A. Denovo 18861 represents k__Bacteria; p__Bacteroidetes; c__Saprospirae; o__Saprospirales; f__Chitinophagaceae. B. Denovo 31743 represents k__Bacteria; p__Firmicutes; c__Clostridia; o__Clostridiales; f__Clostridiaceae; g__Clostridium (Table S10). [file mmc12.zip › Figure S11 041019.png]

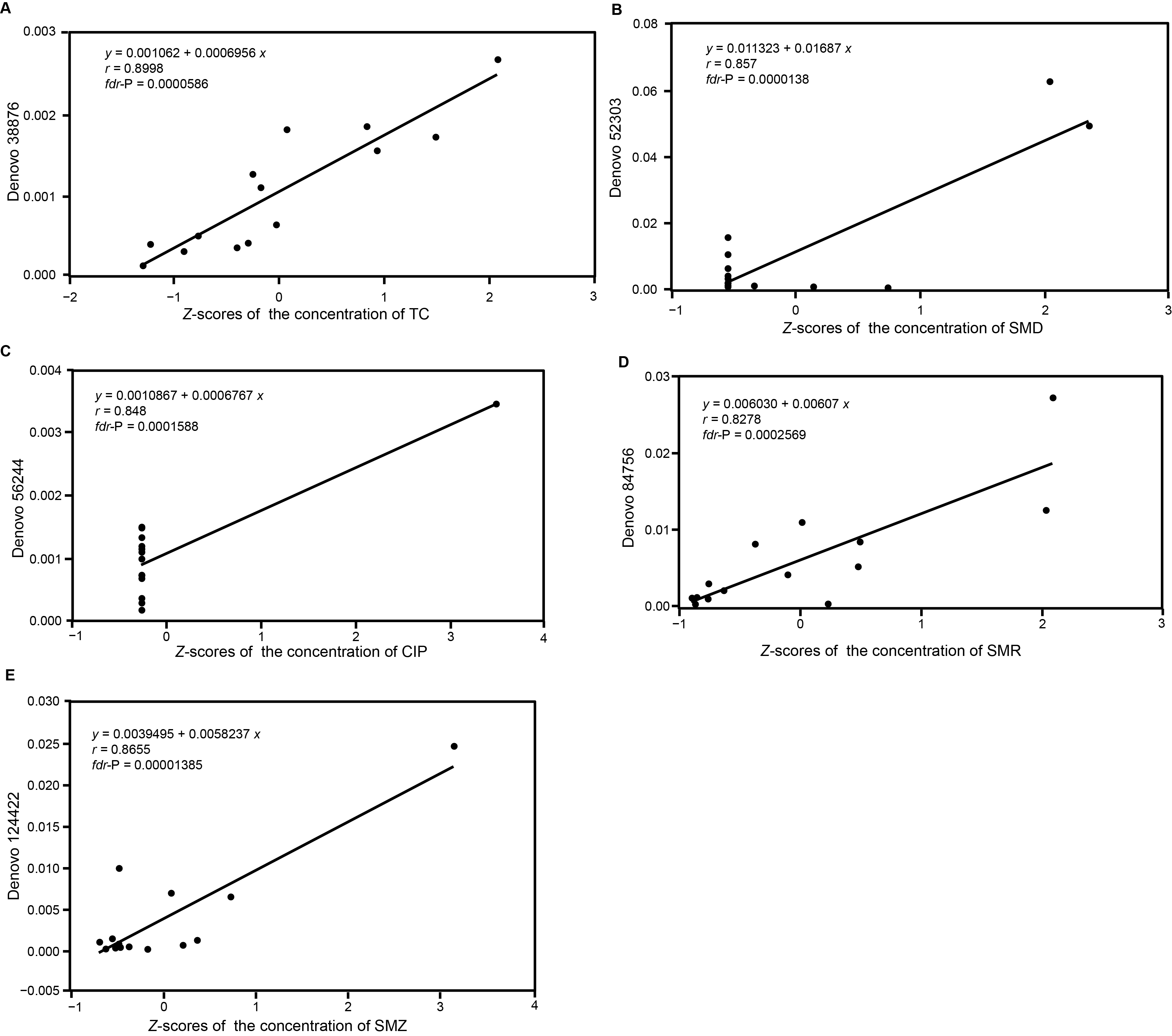

Supplement: Supplementary Figure S12 — The correlation analysis between the relative abundances of OTUs and the concentrations of antibiotics in sediment samples based on Pearson’s Correlation Coefficient with corrected P value Pearson’s Correlation Coefficient analysis was conducted to explore the linear relationship between OTUs and antibiotics, which were transformed to z-scores before calculation, in sediment samples. A. Denovo 38876 represents k__Bacteria; p__Chloroflexi; c__Anaerolineae; o__Caldilineales; f__Caldilineaceae; g__Caldilinea. TC represents tetracycline. B. Denovo 52303 represents k__Bacteria; p__Proteobacteria; c__Alphaproteobacteria; o__Caulobacterales; f__Caulobacteraceae. SMD represents sulfadimidine. C. Denovo 56244 represents k__Bacteria; p__Firmicutes; c__Clostridia; o__Clostridiales; f__Clostridiaceae. CIP represents ciprofloxacin. D. Denovo 84756 represents k__Bacteria; p__Firmicutes; c__Bacilli; o__Bacillales; f__Planococcaceae. SMR represented sulfamerazine. E. Denovo 124422 represents k__Bacteria; p__Bacteroidetes; c__Flavobacteriia; o__Flavobacteriales; f__Flavobacteriaceae; g__Flavobacterium (Table S10). SMZ represents sulfamethoxazole. [file mmc13.zip › Figure S12 041119.png]

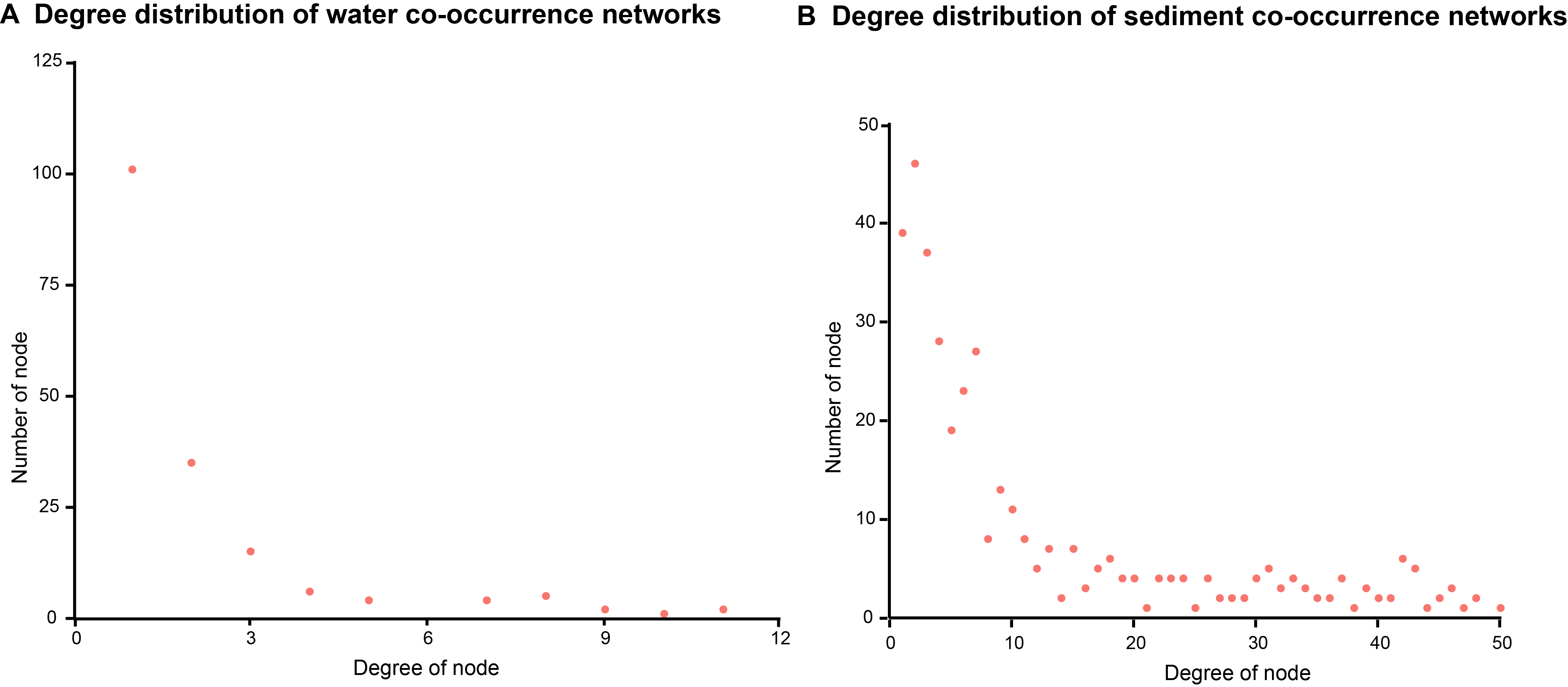

Supplement: Supplementary Figure S13 [file mmc14.zip › Figure S13 041019.png]

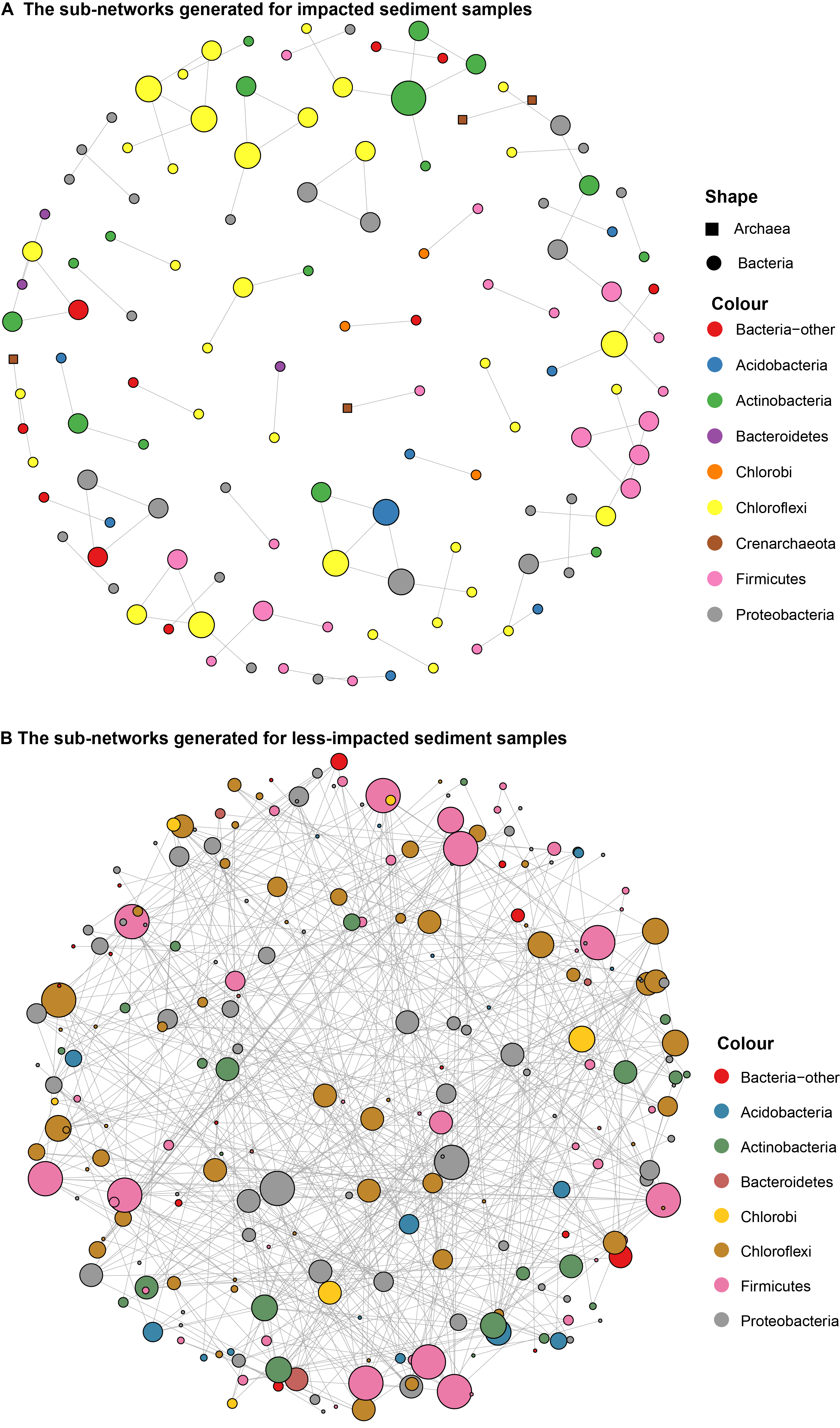

Supplement: Supplementary Figure S14 [file mmc15.zip › Figure S14 041019.png]
